# The role of intracellular interactions in the collective polarization of tissues and its interplay with cellular geometry

**DOI:** 10.1371/journal.pcbi.1007454

**Published:** 2019-11-26

**Authors:** Shahriar Shadkhoo, Madhav Mani

**Affiliations:** 1 Kavli Institute for Theoretical Physics, University of California, Santa Barbara, California, United States of America; 2 Physics Department, University of California, Santa Barbara, California, United States of America; 3 Department of Engineering Sciences and Applied Mathematics, Northwestern University, Evanston, Illinois, United States of America; 4 NSF-Simons Center for Quantitative Biology, Northwestern University, Evanston, Illinois, United States of America; 5 Department of Molecular Biosciences, Northwestern University, Evanston, Illinois, United States of America; University of Pittsburgh, UNITED STATES

## Abstract

Planar cell polarity (PCP), the long-range in-plane polarization of epithelial tissues, provides directional information that guides a multitude of developmental processes at cellular and tissue levels. While it is manifest that cells utilize both intracellular and intercellular interactions, the coupling between the two modules, essential to the coordination of collective polarization, remains an active area of investigation. We propose a generalized reaction-diffusion model to study the role of intracellular interactions in the emergence of long-range polarization, and show that the nonlocality of cytoplasmic interactions, i.e. coupling of membrane proteins localized on different cell-cell junctions, is of vital importance to the faithful detection of weak directional signals, and becomes increasingly more crucial to the stability of polarization against the deleterious effects of large geometric irregularities. We demonstrate that nonlocal interactions are necessary for geometric information to become accessible to the PCP components. The prediction of the model regarding polarization in elongated tissues, is shown to be in agreement with experimental observations, where the polarity emerges perpendicular to the axis of elongation. Core PCP is adopted as a model pathway, in term of which we interpret the model parameters. To this end, we introduce three distinct classes of mutations, (I) in membrane proteins, (II) in cytoplasmic proteins, and (III) local enhancement of geometric disorder. Comparing the *in silico* and *in vivo* phenotypes, we show that our model successfully recapitulates the salient phenotypic features of these mutations. Exploring the parameter space helps us shed light on the role of cytoplasmic proteins in cell-cell communications, and make falsifiable predictions regarding the cooperation of cytoplasmic and membrane proteins in the establishment of long-range polarization.

## Author summary

Planar cell polarity (PCP) is an indispensable and conserved pathway in morphogenesis. In spite of the advances in understanding the different modules of PCP, a comprehensive picture of the intracellular protein-protein interactions necessary for the emergence of long-range tissue polarity is still lacking. In order to address this question, we devised a generalized reaction-diffusion model, through which we investigated the role of cytoplasmic interactions in PCP pathways. The length scale of intracellular interactions is demonstrated to be crucial to the stability of the cytoplasmic segregation of membrane proteins in disordered tissues, as well as the capacity of polarization field for detecting the gradient and geometrical cues. Finally, three classes of mutants are investigated within the context of our model. Comparison with the *in vivo* observations allows us to infer the major contributions of cytoplasmic proteins to the emergence of tissue polarity, and make testable predictions regarding the cooperation of cytoplasmic and membrane proteins in the coordination of collective polarization.

## Introduction

The patterning of an organism requires the coupling of cellular states across multicellular scales. The collective coordination of cellular processes are thus crucial to the emergent phenotype of an organism and requires faithful transduction of directional information across tissues. Planar cell polarity (PCP) is recognized as one of the core mechanisms responsible for such tissue-wide signaling [1–4]. At the cellular level, polarity is defined as the asymmetric localization of two membrane proteins on the apicolateral cell-cell junction. For instance in *Drosophila* wing, two of the core PCP proteins, Frizzled (Fz) and Van Gogh (Vang), are respectively localized at the distal and proximal membranes of each cell. This compartmentalization depends on cytoplasmic segregation of membrane proteins, which is reinforced by intracellular interactions [1–8]. Long-range polarization on tissue-wide length scales is contingent upon intercellular signaling, through which adjacent cells align their polarities. The cooperation of intra- and intercellular interactions to coordinate the long-range planar polarity is the focus of our study [1, 9–11]. The intra- and intercellular interactions are largely carried out via two PCP pathways: “core PCP” and “Fat/Dachsous” [1, 3, 4], each of which involves several interacting membrane-bound and cytoplasmic proteins. Throughout this paper, we adopt core PCP as the reference pathway, according to which we interpret the results. However, the model is constructed based upon phenomenology, general symmetry-based arguments and physical assumptions, hence largely independent of the molecular details of specific PCP pathways.

### Modeling planar cell polarity

Quantitative modeling of PCP and the underlying mechanisms has been of great interest to computational biologists and biophysicists. Several classes of models have been proposed, each focusing on certain aspects of polarity, in particular the subcellular circuitry in charge of single-cell polarity, and intercellular communications that give rise to propagation of polarization over large distances. Nevertheless, the coupling between the two modules has remained a key question. While individual molecular components and their roles vary among different PCP pathways, networks of these components seem to share principal collective functionalities. In addition to the mechanisms of interaction among different components of a PCP pathway, detection of global cues is of great importance, as the direction of polarity is eventually set by such cues. While the coupling of PCP proteins to the cues of chemical origins, i.e. diffusible molecules, is more conceivable, deciphering the readout mechanisms of others such as geometrical and mechanical cues has proven challenging. Therefore, an important question is how each type of these chemical and physical cues couple to the tissue polarity. Some models propose mechanisms through which cells are individually polarized by gradient cues [12, 13]. Others consider scenarios where rotational symmetry breaks spontaneously across the tissue; the long-range polarity is subsequently rotated through coupling with the global cue [14–17]. The second mechanism enjoys high sensitivity and faithful detection of global cues, and is robust against random misreadings of the orientational information by individual cells.

A large class of mathematical models begin with intracellular interactions, which together with cell-cell couplings, give rise to long-range alignment of tissue polarity [13–16, 18]. In the case of vectorial polarity, two types of membrane proteins are considered to interact and localize asymmetrically on cell membranes; their interactions are assumed to regulate the localization of membrane proteins. Intracellularly, the interactions between the membrane-bound proteins are carried out by diffusing cytoplasmic proteins, and are referred to as “nonlocal”, if the diffusion length scales of interaction-mediating cytoplasmic factors are comparable to the cell diameter. Conversely, the interactions are called “strictly local” if the diffusion length scales are much smaller than the cell diameter.

Previous theoretical studies suggest that nonlocality of cytoplasmic interactions is essential to the emergence of long-range polarization [14, 16, 17]. For instance, nonlocal inhibition between opposite complexes is proposed in Ref. [14], as a possible cytoplasmic mechanism for establishing long-range polarity in ordered tissues. In another study, Abley, et.al. [16] considered various possibilities for local and nonlocal interactions between the membrane proteins. Both studies find that the nonlocal interactions lead to the successful cytoplasmic partitioning, and the correlated propagation of polarity over multicellular length scales. Nevertheless, these models predict that global cues are needed for the long-range (tissue-wide) polarity to be achieved within the biological timescales, and conclude that the misorientations in *fat*-mutant *Drosophila* wing is associated with the role of Fat/Dachsous pathway as the putative graded global cue. This proposal does not explain the early margin-oriented polarity of the wing (≲ 17 hours after puparium formation (hAPF), [11]), when *fat* is yet to be expressed [11, 19–21].

However, we shall mention here that the presence of other cues at this stage is not ruled out. Indeed the *Drosophila* wing margin is a source of Wnts. In particular, *wingless* (*wg*) and *wnt4* are expressed at the dorsal-ventral (DV) boundary of the wing disc (the future wing margin), as early as the third instar stage [22, 23]. Together with the fact that Fz can act as a receptor for Wg, it is speculated that the early polarization of the wing is oriented by Wg [7, 23, 24]. Upon eversion of the wing disc, the former DV boundary is converted into the wing margin, where *wg* continues to be expressed throughout wing development [7, 11, 23, 24]. In order for this to be a plausible scenario, the margin-oriented long-range polarity—originally established by Wg in the wing disc—must be robust to cellular stochastic noises in early pupal wing when *wg* expression is limited to the wing margin, i.e. bulk cues are lacking [23]. In Nonlocal Cytoplasmic Interactions (NLCI), we probe the stability of the initially-correlated polarization fields in the absence of cues, by introducing a stochastic noise and measuring the angular dispersion of the cell dipoles. (For brevity, “vectorial polarity of cell” is referred to as “cell dipole” hereafter).

### Molecular ingredients

Core PCP pathway consists of three membrane proteins: (1) seven-pass transmembrane protein Flamingo (Fmi, or its mammalian homolog Celsr), which bind homophilically to form Fmi—Fmi bridges across the cell-cell junctions. (2) Frizzled (Fz), and (3) Van Gogh (Vang, also called Strabismus (Stb)), bind to Fmi on the opposite sides of the cell-cell junctions to form the asymmetric (polarized) complexes [Fz:Fmi—Fmi:Vang] across the cell-cell junctions. Additionally, three cytoplasmic proteins are believed to play crucial roles in intracellular signaling: Dishevelled (Dsh) and Diego (Dgo) bind to Fz, and Prickle (Pk) binds to Vang [1, 3, 5, 8, 25, 26]. While individual cell-cell junctions can be polarized through formation of the Fz:Fmi—Fmi:Vang heterodimers (junctional polarity), in order for a cell to be polarized as a whole, the membrane proteins (Fz and Vang) must be segregated to the opposite sides of the cell. Subsequently, the tissue-wide polarity emerges upon the alignment of the cell dipoles.

Experiments under artificial conditions such as over-expression of membrane proteins, as well as observations of the patterns of polarization near the mutant patches of different cytoplasmic proteins, suggest that although the presence of Dsh, Dgo, and Pk seems to be unnecessary for junctional polarity (less so for small clones of Dsh and Dgo, compared to Pk clones), they are essential to the segregation of the membrane proteins and cellular polarization; thus, their absence impairs long-range polarization [27, 28]. The intracellular interactions and localization of a membrane protein, say Fz, can be upregulated either directly by proteins of the same type, or indirectly by antagonizing the localization of nearby proteins of the other type (Vang in this case). In particular, Fz:Dsh and Vang:Pk are believed to mutually suppress the activities of one another [9, 13, 25, 28, 29]. Regardless of the mechanism, membrane proteins effectively upregulate the localization of proteins of the same type, namely Fz ↔ Fz, and Vang ↔ Vang; and downregulate the localization of the other membrane protein Fz 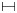 Vang. One of the main goals of this paper is to address the significance of cytoplasmic proteins as the mediators of the interactions between membrane proteins.

### Global cues

Several experimental evidences suggest that in spite of the spontaneous emergence of polarization over multicellular length scales, external cues seem to be necessary for fixing the direction of polarization (see e.g. [1, 3, 30, 31] and references therein for overviews and discussions). The graded distribution of regulatory factors across a tissue [3, 32], mechanical cues [2, 4, 7, 11, 20, 33–35], and geometrical cues [36, 37], are speculated to contribute to the orientational signals, and partially break rotational symmetry. Elongation in particular, has been observed to act as an axial global cue that sets the preferred axis of polarization either parallel or perpendicular to that of elongation, reducing the in-plane rotational symmetry—in each case—to a twofold +/− symmetry. In some cases, elongation gives rise to the polarization of microtubules and vesicle trafficking, which in turn dictates the axis of polarity to be parallel to the elongation [11, 36, 38, 39]. In the mammalian cochlea and skin, polarization emerges perpendicular to the elongation axis [30]. An example is the axial polarity of Celsr1 during the murine skin morphogenesis, where the anterior-posterior polarization is suggested to arise as the tissue is stretched along the medial-lateral axis [37]. Our model intends to provide a mechanistic explanation for the emergence of polarity perpendicular to the elongation axis.

### Geometric disorder

Several studies (e.g. [13, 14, 16, 40]) have proposed underlying physical mechanisms of PCP in *ordered* and isotropic systems. Establishment of long-range polarization during the course of development, however, can precede the formation of an ordered lattice, e.g. margin-oriented polarity in the larval *Drosophila* wing [7, 10, 11]. On the other hand, an experimental study by Ma, et.al. shows that the polarity in mutants lacking graded global cues, is susceptible to local geometric irregularities [20]. While mutants exhibit swirling patterns at the locus of the clone, a sufficiently strong global cue retrieves the wild-type polarization. This observation lends more support to the hypothesis that the tissue polarity is not merely a result of local readout of global cues, but that the orientational order emerges spontaneously from cell-cell communications. The symmetry broken state is characterize, among other quantities, by a correlation length. The correlation length can be estimated, by noting that the distorted patterns of polarity are expected to appear when the clones’ length scales are comparable to that of the polarity correlation length; a very small mutant patch fails to distort the polarization field [20]. Estimation of correlation lengths, in principle, would provide information about some collective behavior of polarization, e.g. sensitivity and response time of polarization upon coupling to a global cue. Therefore, it is important to understand how PCP propagates through geometrically disordered tissues. Note that the above conclusions could also be drawn, with some considerations, from the domineering non-autonomy in core proteins’ phenotypes. The privilege of geometrical mutants is that they are achieved without directly interfering with the PCP pathway, but by the knock-down of *PTEN*. In this paper, the geometric disorder is parametrized by *ϵ*_0_, such that *ϵ*_0_ = 0 corresponds to perfectly hexagonal lattice, and highly disordered tissues can be achieved by tuning *ϵ*_0_ ≳ 0.6.

### Timescales

The polarity pathway involves several dynamical processes characterized by their respective timescales. Incorporating the dynamics of all components not only requires knowledge of the details of molecular interactions and complicates the numerical computations, but also limits the predictive power and applicability of a theoretical model. Therefore, we take advantage of the separation of timescales to abstract the relevant processes from the full complex dynamics. The most important dynamical processes to be considered (particularly in the *Drosophila* wing), are (1) tissue flows and dynamics induced by mechanical stresses, (2) stability of the membrane bound complexes, (3) cytosolic diffusion of the membrane as well as cytoplasmic proteins, and (4) time-dependence of the total amounts of Fz and Vang.

The tissue flows and dynamics of the wing blade take place over ≃ 16 − 28 hAPF, during which the anisotropic mechanical stresses drive oriented cell rearrangements and divisions, that are also accompanied by transient cell shape deformations, and the formation of a rather ordered hexagonal packing of cells [11]. The early margin-oriented polarity is observed to follow these dynamics and rotate to point distally by 28 hAPF. An important point to be noted is that the rotation of cell dipoles, at this stage, does not require the turnover of membrane-bound proteins and formation of new complexes. As a matter of fact, membrane complexes appear to be highly stable over the course of tissue dynamics. The rotation of the polarization field is the result of cell flow, and formation and reorientation of cell-cell junctions, which carry the localized complexes along [11]. While the formation of new junctions and disappearance of old ones decrease the magnitude of polarity during the transient stage, the bound complexes remain stable on the junctions that survive the transition. Thus, insofar as the membrane-bound complexes are concerned, the tissue dynamics does not interfere with the stability of these complexes. In a sense, from the perspective of each cell’s co-rotating frame of reference, the cell’s dipole is oblivious to the tissue dynamics; this particular rotation is not considered to reflect “genuine” dynamics of the polarization field. Focusing on biological interactions, we tend to ignore this stress-induced rotation of polarity. Thus, the concentrations of the membrane-bound proteins seems to vary slowly over timescales of order ≃ 10 (hrs).

The timescale associated with the cytosolic diffusion of proteins can be inferred from the cell sizes and the estimated diffusion constant. The latter is in turn estimated using different methods, either experimentally through FRAP measurements, or theoretically using Stokes’ law for the frictional force on a “spherical” particle (proteins) moving in a viscous fluid (cytoplasm). The diffusion constant from Stokes’ law is found to be inversely proportional to the (Stokes’) diameter *a*_*s*_ of the object. On the other hand, this diameter is related to the atomic mass of the proteins *m*_*p*_ (e.g. Fz, Vang, Dsh, Pk): $a_{s}∼m_{p}^{1/3}$. Assuming that cytoplasm, composed of 70% water, has a viscosity close to that of water, the diffusion constants of core proteins with masses of the order *m*_*p*_ ≃ 10 − 100 (kDa), are in the range of *D*_*p*_ ≃ 0.1 − 10(*μm*^2^/*s*) [41–43] (the values obtained by FRAP, and those from Stokes’ law are often different because of inaccuracies in both methods). The cells in the the wing blade, are around *d*_*c*_ ≃ 5(*μm*) in diameter. The diffusion timescale is then estimated to be $\tau_{\text{diff}}=d_{c}^{2}/D_{p}≃1-10$ (min), roughly two orders of magnitude faster than the dynamics of polarity. Thus, it is safe to assume that the concentrations of diffusing proteins reach steady state, insofar as the dynamics of polarization are concerned.

Finally, the time-variations in the total amount of membrane proteins Fz and Vang are necessary to be estimated, as we will see in Model that the effective rates of binding/unbinding reactions depend on these values. The expression of *fz* in *Drosophila* wing was observed in [44] to be fairly constant over a long time span of about ≃ 24 − 60 hr awp (after white prepupae), roughly corresponding to ≃ 12 − 48 hAPF. On the other hand, the dynamics of polarization in the wing, takes place between ≃ 18 − 32 hAPF. Thus, we believe that although the polarization dynamics is by orders of magnitude slower than the cytosolic diffusion, it is fast enough for the constant concentrations of total Fz and Vang to be a good approximation.

### Terminology

Before introducing the formalism, we shall disambiguate the following terms: “edge” and “junction” are used interchangeably depending on the context emphasizing the geometrical or biological aspects of the problem, respectively. Therefore, one can think of an edge as a cell-cell junction. Next, downregulation and upregulation are sometimes used in lieu of inhibition and activation, respectively. Furthermore, cytoplasmic proteins are sometimes referred to as messenger proteins, to emphasize their role as the mediators of intracellular interactions. Finally, formation/dissociation of cross-junctional complexes are contingent upon the binding/unbinding processes of the membrane proteins on the opposite sides of the junctions. Therefore, the binding/unbinding rates are identical to those of formation/dissociation processes.

### Outline

The objectives of this paper are threefold. We address the role of intracellular interaction in establishing long-range alignment of polarization in tissues with disordered and/or elongated geometries. Varying the characteristic length scales of the upregulating and downregulating cytoplasmic interactions, we explore and unravel the crucial role of nonlocal interactions in the correlations of the polarization field in highly disordered tissues. Furthermore, we demonstrate that the nonlocality of cytoplasmic interactions is of vital importance to (a) the long-range correlations of polarity, and (b) the accurate detection of elongation as a global cue. Finally, to facilitate a conversation between theory and experiment, we investigate our model’s predictions in three classes of *in silico* mutants, and identify phenotypic similarities with experimental observations. The accompanying Supporting Information (S1 Appendix) includes extensive discussions on the assumptions, approximations and the results presented in the Main Text. While the key results are included in the Main Text, interested readers are encouraged to consult the SI for a detailed account of the mathematical definitions, derivations, as well as the secondary results obtained by exploring the parameter space.

## Model

We introduce a set of reaction-diffusion (RD) equations that govern the binding/unbinding reactions of membrane proteins. Each cell is assumed to contain a pool of membrane proteins Fz and Vang, which in their active state bind to the cross-junctional Fmi—Fmi homodimers. The cell-cell signaling occurs when the two types of proteins localize to the opposite sides of the Fmi—Fmi bridges to form asymmetric complexes Fz:Fmi—Fmi:Vang. For the sake of notational convenience, this complex is denoted by F—G hereafter, namely F ≡ Fz:Fmi, and G ≡ Fmi:Vang. The concentrations of total (i.e. bound plus unbound) Fz and Vang, are denoted by *f*_0_ and *g*_0_. These concentrations are assumed to be identical for all cells across the tissue, and constant during the time evolution of polarity. The first assumption is not crucial, should the cell-to-cell variations of concentrations be negligible compared to the average concentrations. As discussed in Introduction, the assumption of time independence could be justified by noting that while the concentrations of localized complexes vary over ≃ 18 − 30 hAPF [11, 18, 28, 42, 45], the level of endogenous Fz has been observed to stay constant over the time span of ≃ 24 − 60 hr awp, which roughly corresponds to ≃ 12 − 48 hAPF [44]. Thus, the time independence of *f*_0_ and *g*_0_ is a reasonable assumption.

In order to covey the concepts concretely, we avoid the notational complexity in this section and simplify the mathematical expressions by adopting a symbolic notation wherever possible. The details are saved for a comprehensive mathematical analysis in Sec. (1) of S1 Appendix. First, *u* and *v* denote the concentrations of the complexes with their F-end and G-end in cell 1, respectively. The complexes can be localized anywhere on the perimeter of the cell. The opposite end of these complexes are naturally in one of the neighboring cells. Note that *v* is not a fundamentally different entity; it is equivalent to *u* calculated in adjacent cells. For clarity, we use *u*_12_ to denote the concentration of *u* complexes localized on the junction separating cells 1 and 2. The equation governing the binding/unbinding dynamics of *u*_12_ reads:

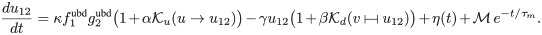
(1)
In the above equation, the first and second terms on the right-hand side represent the rates of binding and unbinding processes, with coefficients *κ* and *γ* characterizing their respective timescales. In the above equation, we have assumed that the *unbound* membrane proteins become uniformly accessible to all points on the perimeter of a cell, before the dynamics of emergent polarization sets in. This assumption requires rapid cytosolic diffusion; much faster than the temporal variations of the junctional concentrations of membrane-bound proteins. As mentioned in the Introduction, the diffusion constant of cytosolic diffusion obtained from FRAP measurements and Stokes’ law, is approximated to be ≃ 0.11(*μm*^2^/*s*) [41–43]. The timescales associated with the diffusion within a cell of characteristic length scale ≃ 5(*μm*) is of the order of ≃ 1 − 10 (min). On the contrary, the concentrations of membrane-bound proteins are observed to be stable over the time span of several hours ≃ 18 − 30 hAPF [11, 18, 28, 45], i.e. at least one order of magnitude slower than the cytosolic diffusion. Therefore, uniform concentration of unbound proteins seems to be a plausible approximation.

In the absence of downregulating and upregulating interactions, the binding/unbinding reaction rates could be expressed in terms of the law of mass action. The formation rate of *u*_12_ on the junction shared by cells 1 and 2 is proportional to the concentrations of unbound F in cell 1 as well as unbound G in cell 2, that are obtained by subtracting the concentrations of bound proteins from the total concentrations;

$$f^{\text{ubd}}=f_{0}-\sum_{⬡}u\;,\;\text{and}\;g^{\text{ubd}}=g_{0}-\sum_{⬡}v.$$ (2)

The symbol ⬡ represents the perimeter of a given cell, where *u* and *v* are nonzero. Note that like *u* and *v*, the concentrations of unbound F and G, *f*^ubd^ and *g*^ubd^ are time dependent.

**Fig 1:**
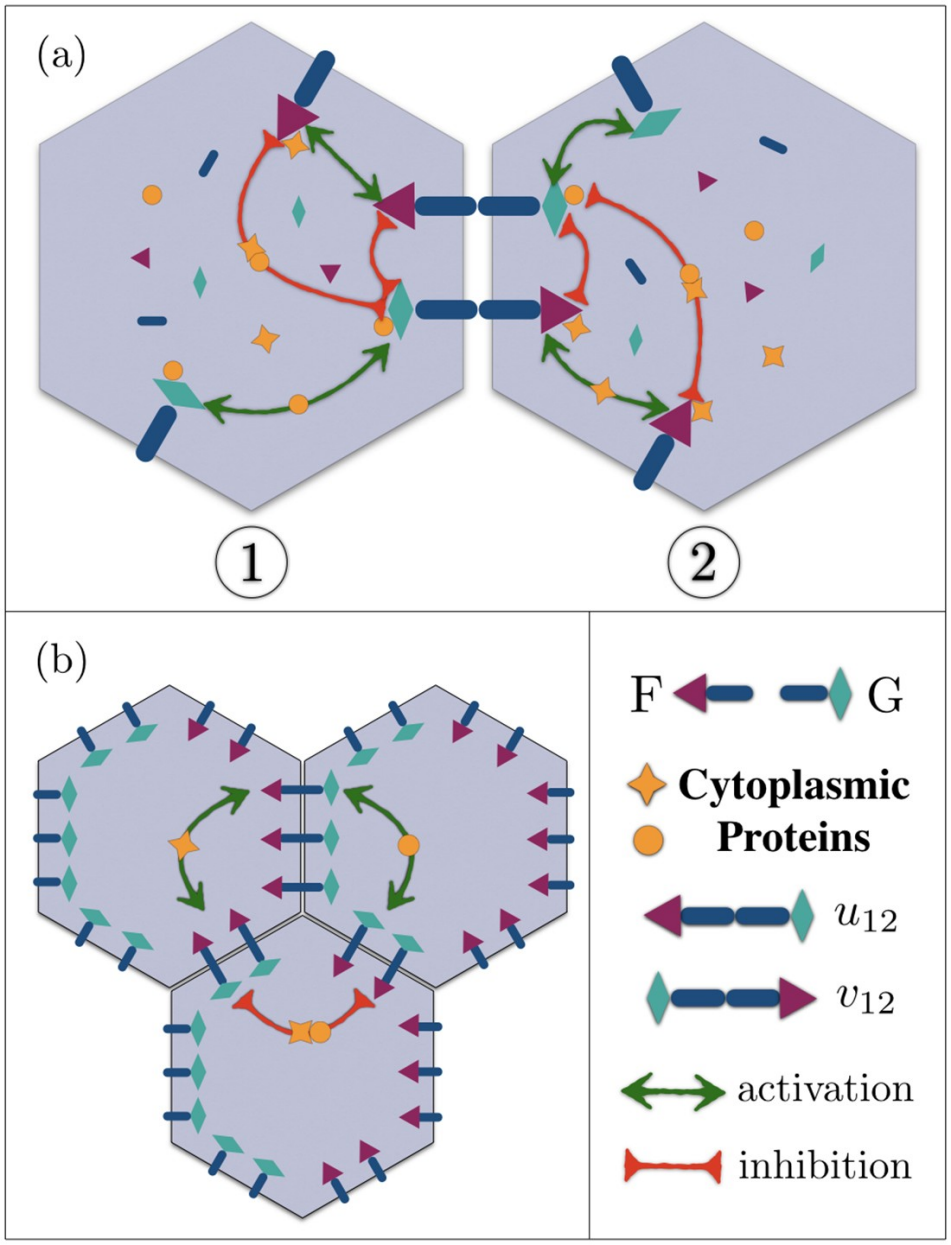
(a) A schematic of intracellular and intercellular mechanisms in adjacent cells. Each cell contains a pool of membrane and cytoplasmic proteins. Membrane proteins Fz (red triangles ▲) and Vang (green diamonds ♦) bind to the transmembrane proteins Fmi (dark blue bars), and form cross-junctional complexes F—G. Two opposite complexes localized on the cell-cell junction are shown in the figure. Nonlocal interactions, illustrated by orange stars ★ and solid disks ●, are mediated by diffusing cytoplasmic proteins that couple the membrane-bound proteins. Star-shaped proteins bind to the red triangles (Fz), get modified and released back to the cytoplasm, to either upregulate the formation of same-polarity complexes, or downregulate the opposite ones; similarly for the orange disks and green diamonds (Vang). While the upregulating interactions between similar proteins, i.e. Fz ↔ Fz, or Vang ↔ Vang, are transmitted by their associated cytoplasmic proteins, stars and disks, respectively, both types of these cytoplasmic proteins participate in the downregulating of the opposite complexes. In order to keep the picture clear, only a few of the pairwise interactions are drawn. (b) shows the protein distributions in a polarized state, where the segregation of F and G is accomplished in each cell through cytoplasmic interactions.

In the second term on the right-hand side of Eq (1), the dissociation rate of *u*_12_ is, besides *γ*, proportional to the local concentration of the same-polarity complexes *u*_12_; the larger the concentration of a *bound* protein, the larger the unbinding rate of the corresponding complex. As discussed previously, in addition to the unassisted reactions, the binding/unbinding processes are augmented by complexes of the same/opposite polarities within each cell, through what we call “cooperative” interactions in this paper. The augmented rates of processes are reflected in the upregulating factor $\alpha K_{u}\left(u\to u_{12}\right)$, and the downregulating factor 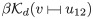. Note that the latter depends on *v*, i.e. the concentration of the opposite complexes. The functional forms of these interactions, $K_{u}$ and $K_{d}$, are introduced in Sec. (1) of S1 Appendix. For now, we only emphasize that these terms represent *nonlocal* interactions between membrane-bound proteins within the *same* cell; that are, in their most general forms, localized on different junctions of a given cell; see Fig (1).

The third term *η*(*t*) on the right-hand side of Eq (1) is a Gaussian white noise: 〈*η*(*t*)〉 = 0, and $\langle \eta \left(t\right)\eta \left(t^{\prime }\right)\rangle =\eta_{0}^{2}\delta \left(t-t^{\prime }\right)$, which arises from the molecular noise of chemical reactions and stochasticity in the upstream signaling pathways. The former is speculated to be the dominant source of noise [14], and is modeled by a Poisson process. The magnitude of noise scales like $\eta_{0}∼1/\sqrt{N_{\text{mol}.}}$, where *N*_mol._ is the number of molecules participating in the binding/unbinding reactions. For the values of the parameters used in our simulations, the magnitude of the stochastic noise is approximated to be *η*_0_ ≃ 0.05 − 0.1; we set *η*_0_ = 0.1, see Table 1.

**Table 1:** List of parameters, variables and their definitions. The parameters with their values mentioned are held fixed throughout the paper. The control parameters, i.e. the geometric disorder *ϵ*_0_, the length scale of the cooperative interactions λ, and the magnitude of elongation E, are to be varied to explore the behavior of polarization in different regimes.

**Parameters**	**Definition**
*f*_0_ = 1	total concentration of F
*g*_0_ = 1	total concentration of G
*κ* = 10	formation rate
*γ* = 1	dissociation rate
*α* = 5	magnitude of cooperative formation
*β* = 5	magnitude of cooperative dissociation
*η*_0_ = 0.1	magnitude of stochastic noise
*ℓ*_0_ = 1	average length of cell-cell junctions
*ϵ*_0_: control param.	magnitude of geometric disorder
λ: control param.	lengthscale of cooperative interactions
E: control param.	magnitude of elongation
**Dynamical Variables**	**Definition**
*u*_*ij*_(*t*) & *v*_*ji*_(*t*)	concentration of [F_*i*_ − G_*j*_]
$\bar{P}\left(t\right)$	magnitude of average polarity
$\bar{Q}\left(t\right)$	average of the dipoles’ magnitudes
O(t)	$\bar{P}\left(t\right)/\;\bar{Q}\left(t\right)$
*ξ*(*t*)	correlation length

Finally, the last term models global cues of chemical origin, which are time dependent in general. We adopt an exponentially decaying functional form with timescale *τ*_*m*_, varying which would capture a broad range of dynamics, from initial (rapidly decaying) to time-independent cues. The exponential decay, however, imposes the decreasing monotonicity constraint, hence excluding the periods when the cues grow in magnitude. Most of the results in the following sections are obtained in the absence of chemical cues (M=0); they are discussed separately at the end of Results. Thus, unless mentioned otherwise, the cues are assumed to be zero.

### Cell polarity

Cell polarity (dipole) is a vector quantity that measures the anisotropy in the distributions of *u* and *v* on the cell perimeter. In other words, polarization of a cell represents the level of segregation of the two types of membrane proteins within the cell. The precise definition of polarity is provided in Sec. (1) of S1 Appendix. Symbolically, cell polarity can be thought of as the difference between the vectors pointing in directions where *u*- and *v*-complexes are localized around the cell:

$$\begin{array}{c} \text{Cell}\;\text{Polarity}∼\;[\to ]-[\leftarrow ]\\ P∼\;\sum_{⬡}\;\;(\;\vec{u}\;\;-\;\;\vec{v}\;). \end{array}$$ (3)

Here, the summation runs over the cell-cell junctions; each junction contributes a vector $\left(\vec{u}-\vec{v}\right)$ pointing towards its center and proportional to the difference between the junctional concentrations of *u*- and *v*-complexes. Cell dipole **P** is defined as the vector sum of these junctional differences (vector sum, because junctional polarities point in different directions). While PCP signaling seems to rely on cell polarities, the notion of polarity can be restricted to individual cell-cell junctions. Keep in mind that cell polarity represents the segregation of membrane proteins that form polarized complexes; thus, equals the vector sum over the junctional polarities. This is important as in the following, we will encounter systems in which the junctions are polarized, yet the vector sum of junctional dipoles renders the cell unpolarized.

### Nonlocal cytoplasmic interactions

Cooperative interactions are mediated by modified/activated cytoplasmic (messenger) proteins. An example of the activation of cytoplasmic proteins is the Wnt-induced phosphorylation of Dsh by Casein Kinase I [46, 47]. Subsequently the messenger proteins diffuse through the cytoplasm to upregulate/downregulate the formation of the complexes of the same/opposite polarities. All pairs of points on the cell membrane are coupled to each other through modifying and emanating the messenger proteins; Fig (1). The magnitude of the coupling is proportional to the concentration of the modified messenger proteins at the position of the target proteins that are localized on the cell membrane. Note that the concentrations of cytoplasmic proteins, unlike those of unbound membrane proteins, are not constant within the cell. The steady-state concentration of a diffusing chemical with diffusion constant *D* and degradation rate *τ*^−1^, is an exponentially decaying function of the distance *r* from the source of the *modified* proteins, namely the position of the membrane-bound *modifying* proteins: $\propto \text{exp}(-r/\sqrt{D\tau })$. Diffusion is inherently a dynamic process. Nonetheless, if the modification and cytosolic diffusion of cytoplasmic proteins are sufficiently fast compared to the time variations of the localized proteins, the concentrations of the messenger proteins reach the steady state before the dynamics of *u* and *v* set in. This assumption is argued to be plausible, by noting that the cytosolic diffusion occurs over timescales of the order of minutes [41, 42], whereas the concentrations of membrane-bound proteins are observed slowly vary over ≃ 10 − 15 (hrs) [11, 28, 45]. Defining the interaction length scale $\lambda =\sqrt{D\tau }$, the magnitude of the cooperative interactions between a pair of points a distance Δ*r* apart, is proportional to exp(−Δ*r*/λ). From a molecular point of view, λ is the length scale a modified protein diffuses before getting degraded. A detailed analysis of cooperative interactions and the assumption of steady-state concentrations can be found in Eq. (3) of S1 Appendix. A few points regarding the nonlocal interactions are in order:

1. It is noteworthy that the exponential form of intracellular interactions is merely a convenient choice with a characteristic length scale; one that is also in accord with a plausible scenario of cooperative interactions mediated by cytosolic diffusion of messenger proteins. Any other function with such properties (e.g. exp(−|Δ*r*|^2^/λ^2^)) would qualitatively lead to the same results as we obtain in this paper.
2. Although there is no *a priori* reason for assigning equal length scales to the upregulating and downregulating interactions, we find it convenient to make this assumption for now. The argument goes as follows: (i) While in actuality the upregulating interactions F ↔ F and G ↔ G, and the downregulating interactions F 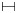 G, are mediated by different cytoplasmic proteins, the way they appear in our model is through interacting complexes F—G. Therefore, the *u* ↔ *u* and *v* 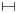 *u* interactions are integrative representations of the actual protein-protein interactions; so is the associated length scale. (ii) In the absence of complete understanding of the molecular details, we assume that both types of interactions are mediated by the same cytoplasmic proteins. Indeed as mentioned above, the upregulating interactions could take place either directly, or indirectly through downregulating the other membrane protein. Later in Nonlocal Cytoplasmic Interactions (NLCI), we relax this assumptions and investigate the effects of unequal length scales.
3. We will see in Local mutations, that the interaction range λ is proposed to be related to the concentration of cytoplasmic proteins. On the other hand, the above definition for λ is independent of concentrations. We emphasize here, that while we defined λ based on some diffusion constant and degradation timescale of cytoplasmic proteins, in the presence of other components, the diffusion constants and degradation timescales are no longer guaranteed to remain the same as their bare values. In other words, the interactions between different molecular components can modify the diffusion parameters, in a concentration-dependent manner.

Before proceeding to discuss the results, we shall make a concrete list of the key model parameters and variables that appear frequently throughout the paper; see Table 1. Some of the parameters are held fixed and their values are included in the table. These values are chosen to satisfy the polarizability condition; discussed in see SI (2.1). The results, however, remain qualitatively insensitive to changing these values, as long as the polarizability conditions are satisfied. There also exist control parameters, the values of which are varied to explore the behavior of the polarization in different regimes. Note that some of the parameters and variables in Table 1 are to be introduced in the following sections.

## Results

In this section we investigate the significance of the nonlocal intracellular interactions in the establishment of long-range polarization, particularly in the disordered tissues. Varying the length scale of the cytoplasmic interactions, we consider two regimes: (1) strictly local cytoplasmic interactions (SLCI), in which λ is negligible compared to cell size, i.e. λ/*ℓ*_0_ → 0; and (2) nonlocal cytoplasmic interactions (NLCI), where λ is of the same order of magnitude as, but smaller than *ℓ*_0_, i.e. λ/*ℓ*_0_ ≲ 1.

The solutions to Eq (1) reveal two distinct mechanisms underlying the emergence of junctional and cell polarities. We find that the former—a prerequisite for the latter—depends on the (relative) abundance of membrane proteins (*g*_0_/*f*_0_), whereas the latter imposes further constraints on the length scale of cytoplasmic interactions (λ). In Sec. (2.1) of S1 Appendix, we show that the necessary and sufficient condition for individual junctions to be polarized is *g*_0_ > g*, where g* depends on other model parameters; it is inversely related to the product *αβ*. For the model parameters in Table 1, the stable junctional polarization appears for *g*_0_ > g* = 0.25. Throughout the Main Text, we set *g*_0_ = 1, well above the threshold; hence the junctions are guaranteed to be polarized.

The asymmetric localization of the membrane proteins on a given junction is a result of the competition between the two types of membrane proteins to occupy the junction. It can be shown that for infinitely abundant membrane proteins the competition has no winner, and the junctional polarization becomes inaccessible. Thus one can think of the finite pools of the two membrane proteins as limiting components, rendering the asymmetric junctional localization of the two proteins a stable state of the system. It is also noteworthy that the finiteness of the total amount of proteins induces an effectively nonlocal interaction, that is implicitly incorporated in our equation. The local interactions between opposite complexes is a functions of their local concentrations. The latter is in turn inversely dependent on the concentrations of the bound proteins on other junctions, since the total amounts of the membrane proteins are finite. Thus, apart from the explicit nonlocal cooperative interactions, a secondary mechanism couples different junctions. This is also reflected in our equations: the reaction equations governing the concentrations of bound complexes on different junctions of a cell are not independent, but rather coupled through the constraint imposed by the total amount of membrane proteins. This indirect nonlocal interaction between the junctions, however, is independent of their distance; in each equation, the proteins’ concentrations on all junctions of the cell appear on an equal footing, irrespective of their relative distances (see Eq (2)). It turns out that the indirect coupling is insufficient for the segregation of membrane proteins, and the polarization of cells.

**Fig 2:**
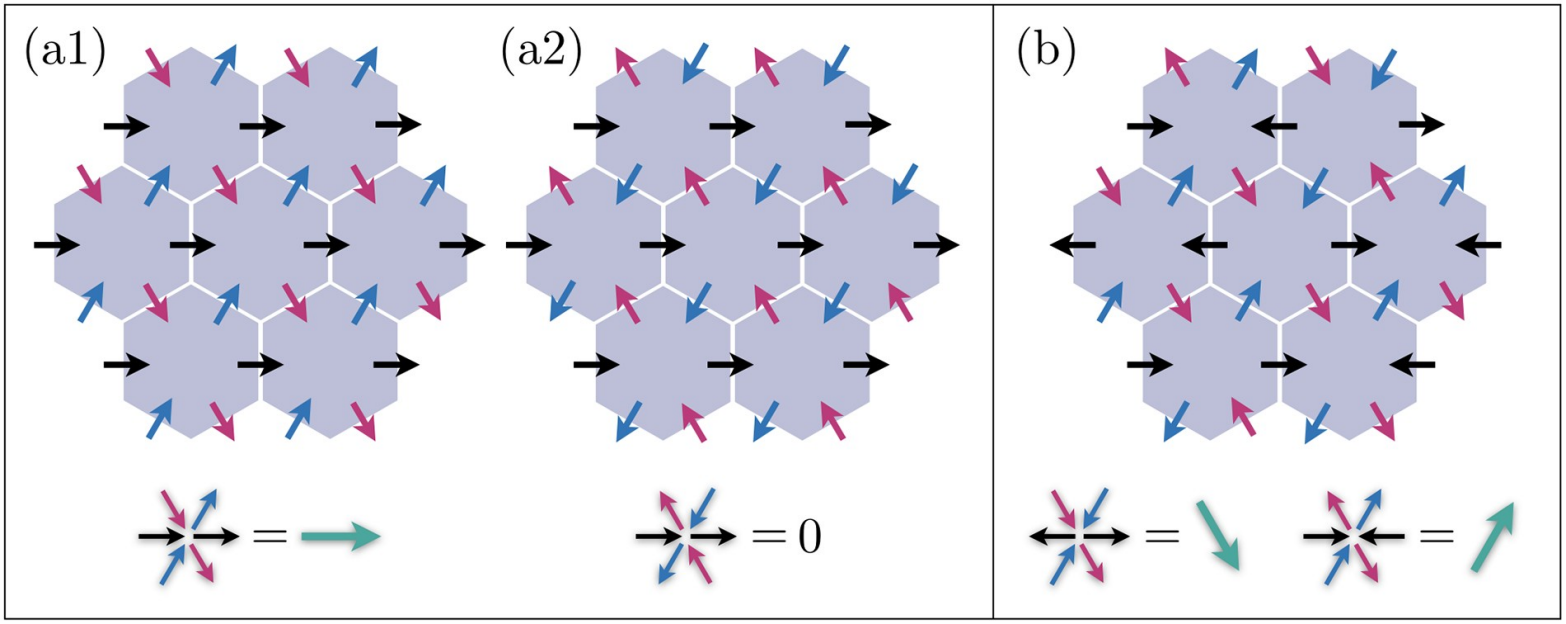
Cartoons of the trivial (a1, a2), and (b) nontrivial patterns. Trivial patterns exhibit uniform polarity across the tissue. While the polarization pattern in (a1) is highly stable, the one in (a2) is immediately destabilized by slightest nonlocal cytoplasmic interactions. In (b) all junctions are polarized with equal magnitudes, yet the cell dipoles are oriented randomly with little correlation; two dipoles belonging to the central cell and its right neighbor are shown.

In order to unravel the richness and complexity of the possible polarization patterns, we illustrate three distinct configurations in Fig (2). The left panel shows two special patterns of polarization in (a1) and (a2), where the cell dipoles are identical across the tissue; the former is a cell-polarized state whereas the latter has zero-net polarization. We call these “trivial” patterns. (a1) shows a perfectly polarized state where the segregation is fully accomplished. The configuration in (a2), in principle satisfies Eq (1); a configuration where the cells, unlike edges, remain unpolarized. However, the polarity becomes highly unstable, as soon as the nonlocal interactions come into play (see Sec. (2.2) in S1 Appendix). Note that in Eq (1), the second term inhibits the localization of *u* in the vicinity of *v* (and vice versa). The configuration in (a2) is the most intermixed pattern, where adjacent junctions carry alternating polarities, and the segregation is far from complete. Therefore, even if the initial distribution of proteins corresponds to such polarity patterns, slightest stochasticity suffices to destabilize the initial state, and redistribute the proteins to form a polarized state. The right panel, Fig (2b), illustrates an irregular pattern in which the polarity differs from cell to cell; we call these patterns “nontrivial”. Below, we show that these states appear when the cytoplasmic interactions are local. Cytoplasmic segregation is incomplete in these states, and the polarization is characterized by incoherently oriented dipoles; hence no long-range correlation.

### Global measures of correlation

In addition to the correlation function which is defined rigorously in Sec. (1) of S1 Appendix, two global quantities are introduced that help us gain insight into the angular correlation of polarization. Earlier in the Model we introduced cell polarity, which is a vector quantity. Averaging cell dipoles over the entire systems is defined as the global tissue polarity. We denote the *magnitude* of this vector by $\bar{P}$. On the other hand, one could *first calculate the magnitude* of each cell dipole, and *then average* over the *magnitudes*; we call this $\bar{Q}$. The latter is guaranteed to be larger than the former per definition: $\bar{Q}$ is an average over positive values (magnitudes of dipoles), whereas $\bar{P}$ is the magnitude of a vector which is in turn obtained by averaging over cell dipoles, and could in principle be very small in size. The ratio of the two quantities $O\equiv \bar{P}/\bar{Q}$ is bounded from above by one.

It is pedagogical to consider the following two limits: (1) Randomly oriented dipoles: the average over the dipoles returns a vector with nearly zero magnitude $\bar{P}\to 0$, whereas the magnitude of individual dipoles are not necessarily small, and so is their average $\bar{Q}↛0$, thus $O=\bar{P}/\bar{Q}\to 0$. (2) Perfectly aligned, identical dipoles across the system (Fig (2a1)): the average is a vector with both direction and magnitude equal to those of a single dipole. The average of magnitudes also equals the magnitude of a single dipole, and $O=\bar{P}/\bar{Q}\to 1$. Therefore, O provides an intuitive global measure for the long-range order of the tissue polarization. We shall emphasize, however, that while O→1 guarantees perfect alignment, the opposite limit O→0 does not necessarily imply complete loss of order. There exist intermediate configurations where polarity is ordered over medium-size patches of cells, yet averaging over the system makes O small. This brings us to the more informative measure of order, the correlation length. Denoted by *ξ* in the following sections, correlation length characterizes the length scales over which the polarity retains the angular correlation. See Sec. (1) in S1 Appendix for precise definition of correlation length.

Below we discuss the results of our numerical simulations in tissues with strictly local and nonlocal interactions. The discussion is followed by the investigation of the role of cytoplasmic interactions in elongated tissues. Save for the stability analysis of polarization, the *initial* distributions of F and G on the cell membranes are assumed to be random in all simulations.

### Strictly Local Cytoplasmic Interactions (SLCI)

**Fig 3:**
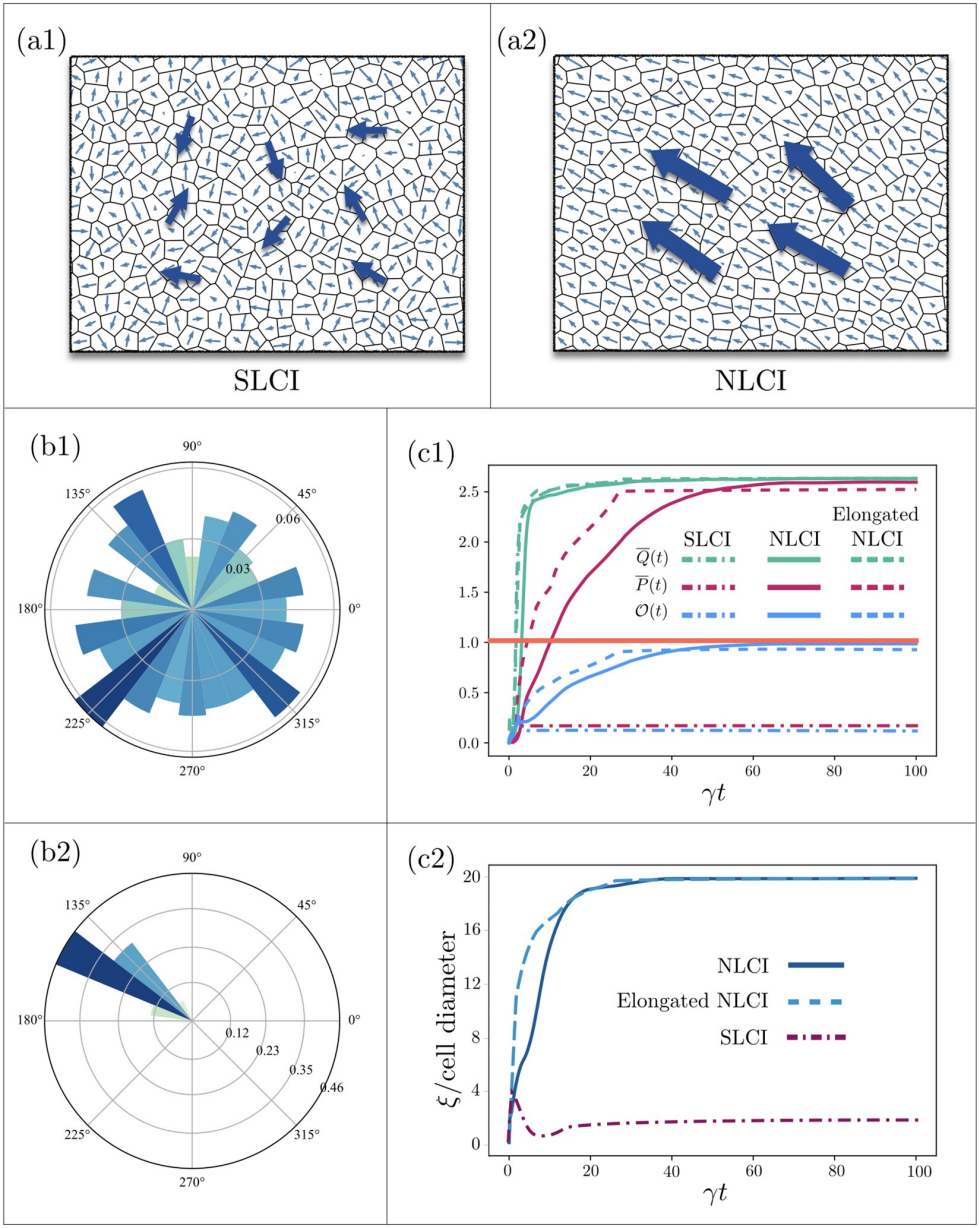
Generic steady states of two systems in the SLCI (λ/*ℓ*_0_ = 0.01) and NLCI (λ/*ℓ*_0_ = 0.5) regimes are shown in (a1) and (a2), respectively. Cellular geometry, initial conditions, and model parameters (other than λ) are identical in (a1) and (a2). The big arrows show the average direction of dipoles over patches of a few cells. Angular distributions of the dipoles in the two cases are shown in (b1) and (b2), which reveal the failure of SLCI in establishing long-range polarization. Time evolutions of $\bar{Q}$, $\bar{P}$, and O, for isotropic systems with SLCI (dash-dotted lines), with NLCI (solid lines), as well as elongated systems with NLCI (dashed lines) are shown in (c1). Correlation lengths are illustrated in (c2); unlike in the NLCI case where the angular correlations extend over tissue scales, the correlation length in the case of SLCI remains limited to a few cell diameters. For clarity, the small fluctuations of all the curves, induced by stochastic noises, are removed through ensemble averaging.

A generic steady state of the systems with local cytoplasmic interactions, and the rose-plot of the angular distribution of dipoles are shown in Fig (3a1) and (3b1), respectively. Big arrows in (a1) represent local averages of polarity on patches of a few cells, which exhibit little angular correlation. The dash-dotted curves in Fig (3c1) and (3c2) demonstrate the time evolution of $\bar{Q}\left(t\right)$, $\bar{P}\left(t\right)$, their ratio O(t), as well as the correlation length *ξ*(*t*), respectively. To facilitate the comparison, Fig (3c1) and (3c2) include the corresponding quantities computed for other cases of study to be discussed in Nonlocal Cytoplasmic Interactions (NLCI) and Polarization in elongated tissues. Comparing $\bar{Q}\left(t\right)$ and $\bar{P}\left(t\right)$, we see that while cells can develop nonzero dipoles, they fail to align; the dipoles add up to nearly zero when averaged over patches of 2 cell diameters and larger. This can be deduced from the SLCI correlation length in Fig (3c2).

Given the lack of long-range polarization in this limit, one can consider SLCI and the associated phenotypes to be a mutation that is a consequence of disrupted cytoplasmic interactions. Note that the assumption of steady-state concentrations of messenger proteins relies on the abundance of modified cytoplasmic proteins, which is in turn dependent on the amount of unmodified (yet-to-be-modified) proteins. Thus under-expression of cytoplasmic proteins and/or reduced rate of modification would impair the transmission of nonlocal interactions.

### Nonlocal Cytoplasmic Interactions (NLCI)

Nonlocal interactions, as introduced in Model, are mediated by diffusing cytoplasmic messenger proteins. As mentioned previously, in the presence of nonlocal interactions, the adjacent edges of a cell prefer to carry the same polarities; nonlocality renders the alternating junctional polarities an unfavorable configuration and destabilizes trivial solutions with zero-net polarity depicted in Fig (2a2). Therefore, each cell tries to segregate the two membrane proteins to the opposite sides. This intracellular mechanism, accompanied by the intercellular interactions, conspire to align the dipoles on patches the grow in time over progressively larger distances.

Fig (3a2) shows a typical configuration of the steady-state polarization for a system in the NLCI regime. The range of nonlocal interactions is set to λ/*ℓ*_0_ = 0.5; chosen to maximize the alignment, and is obtained by evaluating the angular correlation for λ in the wide range of 0.01 ≲ λ/*ℓ*_0_ ≲ 0.8. The results and discussions are included in Sec. (2.3) of S1 Appendix. Interestingly, the correlation exhibits a non-monotonic behavior with respect to λ. Starting from strictly local interactions, the correlation length increases initially with λ, and peaks at λ/*ℓ*_0_ ≃ 0.5 − 0.6 —the exact value depends on indeterministic factors including the specific geometry of the tissue. For λ/*ℓ*_0_ ≳ 0.6 the correlation drops. The reason is that if the diffusion length scale of the messenger proteins is comparable to the diameter of the cells, a complex on one side of a cell would upregulate the formation of the complexes of the same polarity, not only in its vicinity, but also on the opposite side of the cell. This upregulation impedes the segregation which is meant to gather all complexes of a specific type on one side of and push the opposite complexes to the other side of the cell. Therefore, as λ/*ℓ*_0_ exceeds ≃0.6, the complexes begin to colocalize and distribute evenly around the cell. Interestingly this regime appears in one of the classes of local mutants (type III) discussed below.

We focus on λ/*ℓ*_0_ = 0.5 hereafter. The dynamics of $\bar{Q}$ and $\bar{P}$, shown in Fig (3c1), imply that the emergence of collective polarization—from random initial distribution and in the absence of a global cue—consists of two distinct stages: (i) the segregation of PCP proteins within each cell, and saturation of the magnitude of cell polarity, accompanied by (ii) formation of polarized local domains, which is followed by coarsening and alignment of the domains across the tissue. The first and second stages are carried out mostly through intracellular and intercellular interactions, respectively.

### Unequal length scales of interactions (λ_*u*_ ≠ λ_*d*_)

We recall from Model that the characteristic length scale of upregulating and downregulating interactions were argued to be of the same order of magnitude. Here, we further investigate the role of nonlocality in cytoplasmic interactions, by varying the two length scales independently.

Borrowing the terminology from the well-known mechanism of local activation—nonlocal inhibition (LA-NLI), we introduce four combinations of the relative activation-inhibition length scales: (i) LA-LI, (ii) LA-NLI, (iii) NLA-NLI, and (iv) NLA-LI. The LA-LI and NLA-NLI cases are essentially the same as SLCI and NLCI, respectively, which are already addressed. Mechanism (iv) is incapable of establishing polarity, since the long-range activation hampers full segregation. The most interesting case to be discussed is the second scenario where activation, unlike inhibition, acts locally. To isolate the effects of the variations of the activation length scale, the cellular geometries and the initial distributions of membrane proteins are held fixed for all values of λ_*u*_. Note that the downregulating interactions remain nonlocal; λ_*d*_/*ℓ*_0_ = 0.5.

The summary of our results are as follows: For tissues with small geometric disorder, the two mechanisms of LA-NLI and NLA-NLI work equally well, and both guarantee the long-range polarization. Upon cranking up the geometric disorder beyond *ϵ*_0_ ≃ 0.5, the angular correlation arising from NLA-NLI is arguably larger than that of LA-NLI. The results of our simulations reveal an interesting role of geometric disorder, and the necessity of nonlocal activation for long-range polarity to emerge in highly disordered systems. This is crucial to understanding the PCP in systems where long-range polarity appears before the formation of ordered tissue, an example of which is the margin-oriented polarization in *Drosophila* wing, when the wing blade is highly disordered. See Sec. (2.3) in S1 Appendix for detailed analyses and graphs.

### Mechanism of cytoplasmic segregation

A crucial point to be discussed is in regard with the level of segregation. Naïve interpretation of $\bar{Q}$ would suggest that the absence of segregation prohibits large values of $\bar{Q}$, since the cells remain unpolarized. Nonetheless, as we see in (c1) for local interactions, $\bar{Q}$ grows and asymptotes at the same value as that of nonlocal interactions, which is claimed to be the right mechanism for the segregation to be accomplished. We elaborate on this issue in Sec. (2.3) of S1 Appendix, and show that while $\bar{Q}$ acquires large values, the cell-to-cell variations of the magnitude (and direction) of polarity is large, namely this quantity merely represents the average of the magnitudes. In NLCI regime, however, the value of $\bar{Q}$ is large due to the nearly coherent and complete segregation of membrane proteins in each cell, and the variation of dipoles among cells is minuscule. In summary, the segregation in tissues with local interactions is not coherent, and arises in some cells due to initial distribution of proteins. Nonlocal interactions amount to the redistribution and coherent segregation of proteins across the tissue. For a thorough discussion we refer the reader to Sec. (2.1) of S1 Appendix.

### Stability analysis

**Fig 4:**
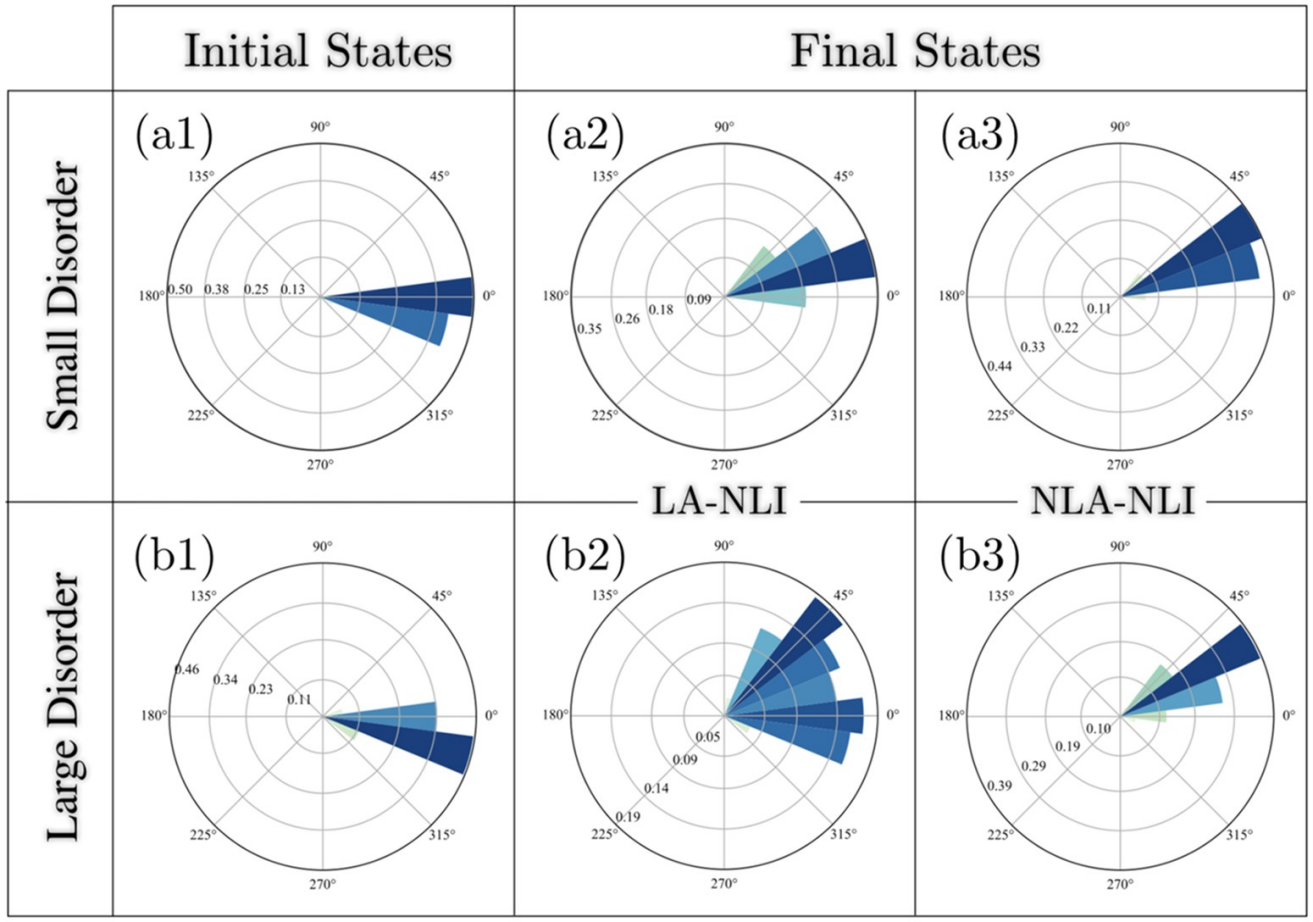
Comparison of the polarization stability in systems with local and nonlocal upregulating interactions, but identical cellular geometries and initial conditions (for each value of the geometric disorder). The magnitude of stochastic noise equals *η*_0_ = 0.1 in all cases. (a1) illustrates a polarized initial condition in a tissue with small geometric disorder *ϵ*_0_ = 0.45. (a2) and (a3) show the steady-state angular distributions of dipoles in the same tissue with LA-NLI and NLA-NLI mechanisms, respectively. The same quantities are depicted in (b1), (b2) and (b3), but in tissue with large geometric disorder *ϵ*_0_ = 0.6. Loss of angular coherence in (b3), indicates that the absence of nonlocal upregulating interactions makes polarity susceptible to stochastic noises in disordered tissues.

We examine the stability of polarization against stochastic noise, and compare the responses of the polarization in tissues with local and nonlocal upregulating interactions. In order to highlight the detrimental effects of geometric disorder in the absence of nonlocal upregulating interactions, this comparison is repeated for different levels of geometric disorder. Fig (4) demonstrates the results for small (*ϵ*_0_ = 0.45), and large geometric disorder (*ϵ*_0_ = 0.6). To isolate the effect of upregulating interactions, the initial conditions and tissue geometries are fixed. The steady-state angular distributions of the dipole are shown in (a2, a3), and in (b2, b3) for small and large disorders, respectively. Comparing the final distributions we realize that unlike in the nearly ordered tissues, local activation fails to guarantee the long-range alignment of cell dipoles in highly disordered tissues; the latter is more susceptible to stochastic noises, and loses the initially imposed polarity. This effect becomes progressively more pronounced as the geometric disorder increases (compare (a2) and (b2)). Note that even in tissues with nonlocal activation, the final polarities are rotated compared to the initial state. This is due to the bias provided by the irregular geometry, and the large stochastic noise that drives the polarization away from the initial state to the stable state dictated by geometry. Nonetheless, the angular correlation of the polarization is preserved. The role of geometric disorder in rotating the polarization becomes evident by noting that the polarization retains its initial direction in ordered systems.

This result should be acknowledged in light of the experimental observations of the margin-oriented Fz/Vang polarity in early pupal wing of *Drosophila* (≲ 16 hAFP) [11]. This stage of the wing development resembles the circumstances under which we probed the polarization stability in this section: (a) the putative early bulk cue, provided by Wg, is confined to the margin, and *fat* expression has not started yet. Therefore the dipoles are initially aligned, but are no longer subject to a cue. (b) The mechanical stress in the wing blade has neither established the geometric order in the tissue, nor has it rotated the dipoles, hence not acting as a cue either. Therefore, we have a long-range polarization field subjected to stochastic cellular noises, in the absence of global cues, and in a highly disordered tissue. Based upon the stability analysis in highly disordered tissues, our model predicts that the local activation is insufficient for maintaining the polarization, and the cytoplasmic upregulating interactions are required to be nonlocal to survive the stochastic noises.

### Directional cues

We consider two types of cues: bulk and boundary signals, each of which may be persistent or transient. Bulk cues are assumed to have constant gradient and couple to the F component across the entire tissue. Boundary cues couple only at the boundaries, and are modeled as a strong polarizing signal that effectively maintains the polarity of a few layers of cells into the bulk (resembling the Wg pattern in the margin of the early pupal wing). The magnitude of the bulk gradient cues is in general time dependent; see Eq (1). At *t* = 0 the slope of the gradient equals M and drops exponentially in time over a timescale *τ*_*m*_. Large *τ*_*m*_ corresponds to persistent cues, whereas *τ*_*m*_ → 0 models an initial cue that disappears rapidly. Before proceeding, we note that there exist two timescales in this analysis: the timescale of polarization dynamic, and the persistence timescale of the cue *τ*_*m*_.

The results in a nutshell are as follows. (1) The response of the polarization field to the global cues reveals that nonlocal cytoplasmic interactions enhance the sensitivity of the collective polarization to the gradient of global cues, and that the reorientation of dipoles requires weaker gradients in systems with nonlocal interactions, compared to those with local interactions. (2) The initial slope of the gradient required for the signal to be detected was found to grow as the cues decay more quickly. (3) In accord with the former observations, in comparison to the systems with nonlocal interactions, the detection of cues occurs over exceedingly larger timescales in systems with SLCI. The detection of transient cues was shown to be feasible in a study by Fischer et. al. [18], wherein the hitherto proposed theoretical PCP models were investigated. (4) In the case of boundary cues, in *nearly ordered* tissues with *infinitesimal* stochastic noise, local interactions suffice to detect the signal. Presence of geometric disorder and/or stochastic noise, however, necessitates nonlocality of cytoplasmic interactions for the dipoles to align with the cue. Finally, an interesting observation is that, (5) nonlocal interactions appear to detect sufficiently large *initial boundary* signals. The latter is implemented by polarizing a column of cells with significantly larger asymmetry of proteins’ distributions (i.e. larger dipoles). The imposed condition is relaxed immediately after the onset of dynamics. This observation implies that a temporary boundary signal would, in principle, be able to rotate the dipoles, provided that the cytoplasmic interactions are nonlocal. Given that the onset of polarization alignment precedes the tissue ordering [10], the nonlocality of cytoplasmic interactions seems to be of vital importance to the faithful detection of directional cues.

### Polarization in elongated tissues

Elongation has been speculated and observed to act as a global cue that couples to PCP proteins in some systems, e.g. *Drosophila* wing, mammalian cochlea and mice medial-lateral skin [37]. Assuming that a set of signaling proteins are capable of detecting the geometric information, stretching a tissue would rotate the dipoles either parallel or perpendicular to the axis of elongation. This is a purely symmetry-based argument and is independent of the pathway-specific molecular details. In Ref. [37], the authors observe that the medial-lateral stretching of the mice skin gives rise to anteroposterior polarization. Furthermore, it was shown in the same study that the perpendicular polarization is not due to a naïve incorporation of length in the definition of polarization, but that the short junctions are indeed depleted of proteins. Here we show that NLCI, through increasing the strength of the cooperative self-interactions, enhance the stability of F-G complexes on longer junctions. Intuitively, unbound proteins receive, on average, stronger signals from complexes localized on longer junctions; see Sec. (3) in S1 Appendix. In S6 Fig, we plot, for different λ’s, the dependency of self-interactions on the edge lengths. The magnitude and angle of elongation are denoted by E and *ϕ*^*e*^, respectively. The former is defined such that E=0 for isotropic tissues, and the latter is measured from the *x*-axis.

**Fig 5:**
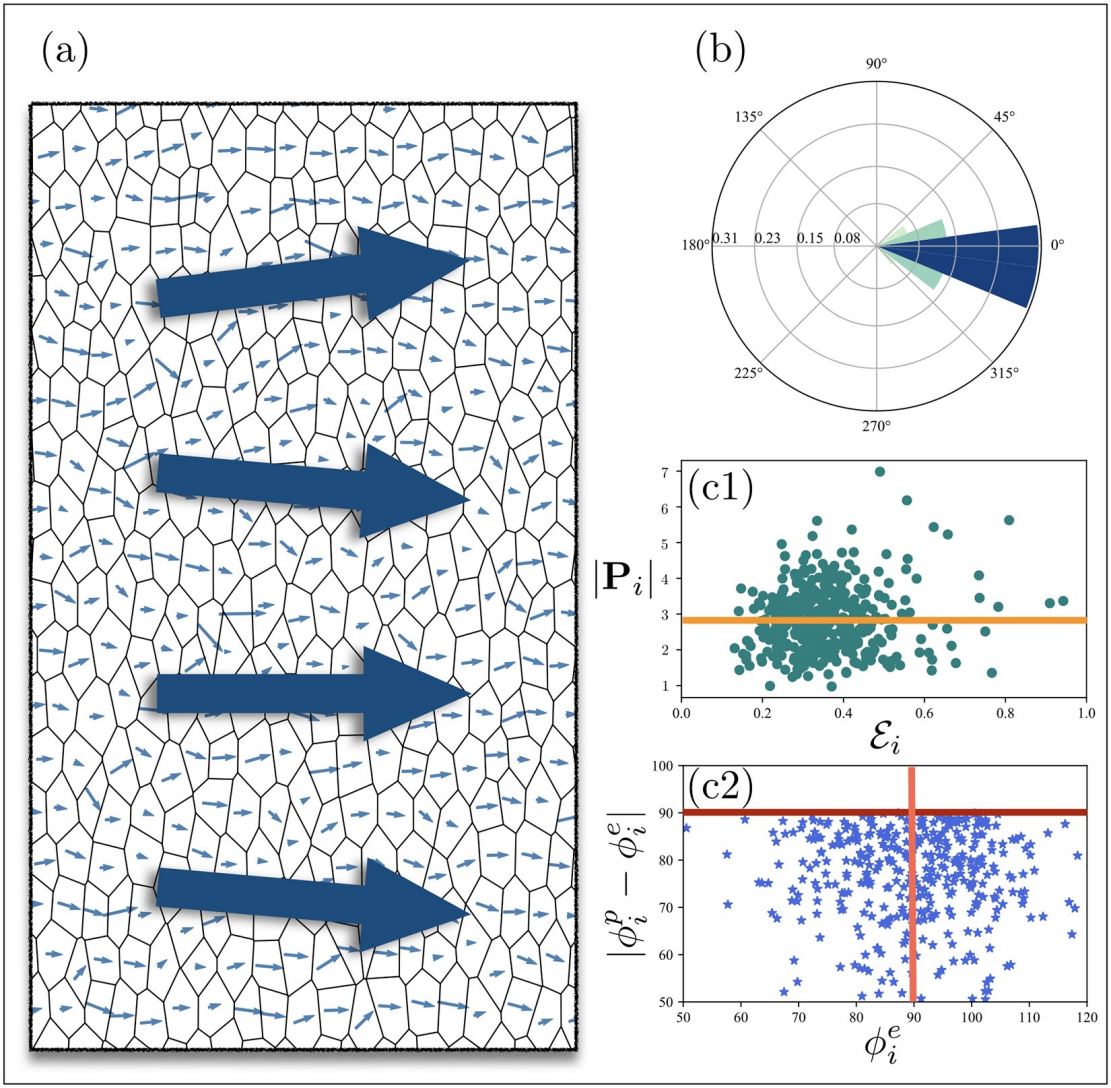
(a) and (b) represent the final configuration and angular distribution of polarization in an elongated tissue with E=0.4. Cell-by-cell magnitudes of the dipoles |**P**_*i*_| versus the magnitudes of elongations $E_{i}$, are shown in a scatter plot (c1). The orange line marks the mean magnitude of the cell polarities, $\bar{Q}$. In (c2) the relative angles of the cell dipoles and elongations $|\phi_{i}^{p}-\phi_{i}^{e}|$, are plotted against the angles of the cell elongations. The average angle of elongation, marked by the vertical red line, is 90° with respect to the *x*-axis. By definition, the relative angles $|\phi_{i}^{p}-\phi_{i}^{e}|$ fall below 90° (horizontal dark red line).

Time evolution of $\bar{Q}\left(t\right)$, $\bar{P}\left(t\right)$, O(t) and *ξ*(*t*) are shown in Fig (3c1) and (3c2). Fig (5b) illustrates the steady-state of the polarization field in an elongated tissue with E=0.4. In order to see (a) whether the observed polarity is a collective effect or is due to single-cell geometry, and (b) that polarity is not a trivial geometrical effect, we make the cell-by-cell scatter plots of the magnitudes and angles of the cell dipoles, |**P**_*i*_| and $\phi_{i}^{p}$, vs. the magnitudes and angles of the cell elongations, $E_{i}$ and $\phi_{i}^{e}$; see Fig (5c1) and (5c2). The infinitesimal cell-by-cell correlation between |**P**_*i*_| and $E_{i}$ indicates that the perpendicular polarization is not due to the naïve definition of polarity based on junctions’ lengths. Furthermore, lack of correlation between the angles of cells’ elongations $\phi_{i}^{e}$ and dipoles $\phi_{i}^{p}$, reflected in (c2), implies that the perpendicular polarization in elongated tissues is not a local, but a collective effect. Note that if the two angles were correlated, the difference $|\phi_{i}^{p}-\phi_{i}^{e}|$ would be close to 90° for all cells, regardless of their angle of elongation; namely all points would collapse on the horizontal red line.

### Local mutations

Studying the mutant phenotypes is a common approach for identifying the functions of the corresponding proteins. Local PCP mutations are characterized by irregular patterns of polarity, which might be induced autonomously and/or non-autonomously by mutant clones [3, 6, 48–50]. Here, we introduce three distinct classes of mutations within the context of our model, involving (I) membrane proteins, (II) cytoplasmic proteins, and (III) local geometric disorder. Comparison with the *in vivo* counterparts helps us uncover the roles of individual components in the PCP pathway. For instance, a trivial correspondence between Vang and G is deduced by comparing the *in vivo* phenotypes of *Vang*^−^, and the *in silico* phenotypes associated with small *g*_0_. Unsurprisingly, our model successfully captures the major functionalities of membrane proteins in the core PCP pathway. The main challenge is to infer those of the cytoplasmic proteins.

### Inducing *in silico* mutations

**Fig 6:**
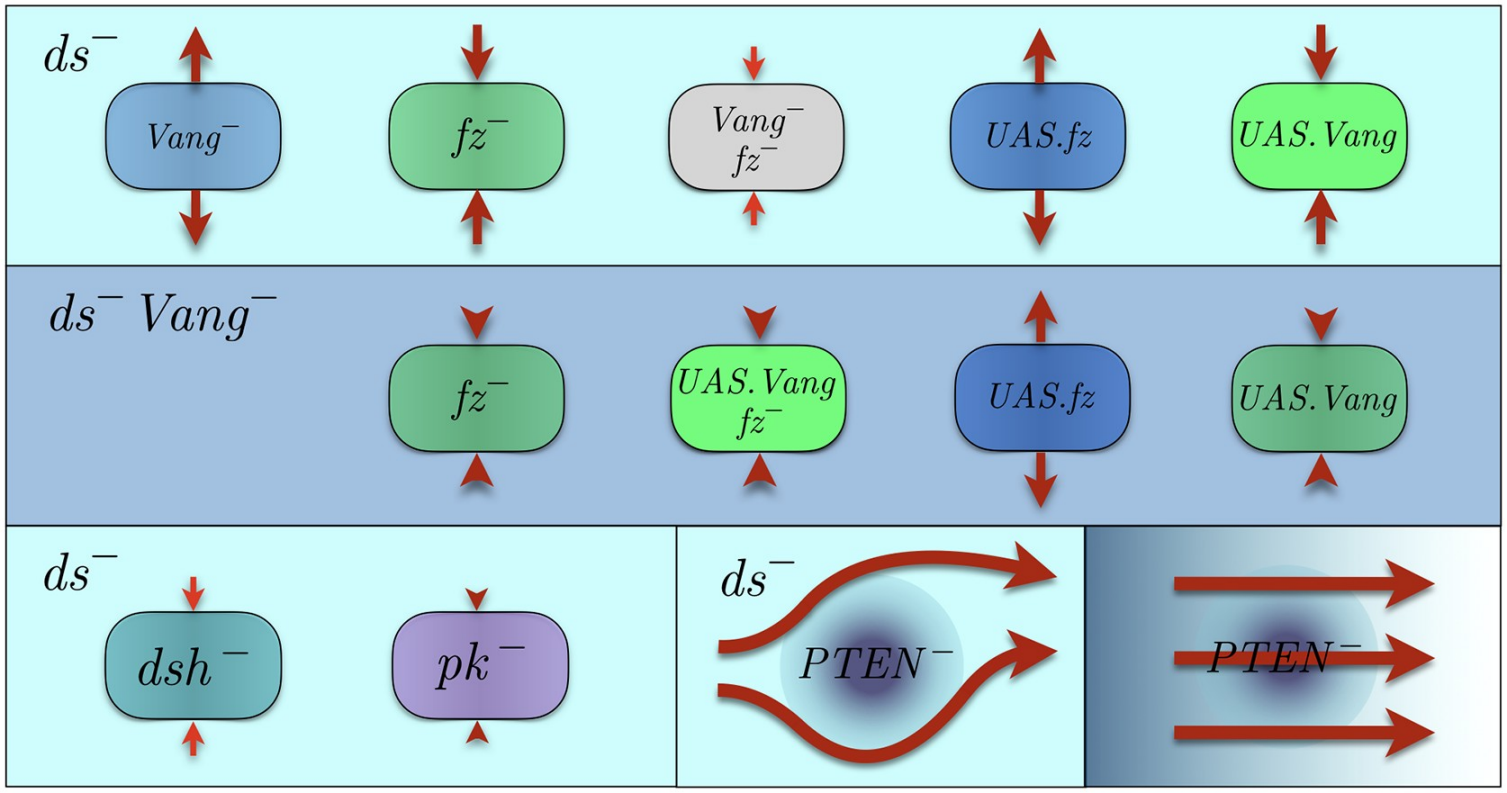
First and second rows illustrate various clones embedded in *ds*^−^ and *ds*^−^ *Vang*^−^ backgrounds, respectively; *ds*^−^ implies the absence of global cue. Since Fz and Vang appear on an equal footing in the model, the background mutants are only shown for *Vang*^−^ tissues. The corresponding phenotypes in *fz*^−^ background, are obtained by replacing Fz and Vang and flipping the arrows. The colors of the background and clones are chosen as follows: blue and green represent Fz and Vang, respectively. Together, Fz and Vang make the cyan background in the first row. Lack of either one is represented by the complementary color. The left panel of the third row shows mutants lacking cytoplasmic proteins in *ds*^−^ background. Red arrows must be interpreted as the directions of the polarity distortion measured with respect to the wild-type polarity; not the direction of the resulting polarity (see S7 Fig for further clarification). The thickness and length of an arrow indicate the magnitude and spatial extension of non-autonomy, respectively. The right two panels of the third row correspond to the geometrically disordered clones, with and without global graded cues. In this case the arrows show the resulting polarization fields.

Total concentrations of the membrane proteins are represented by *f*_0_, *g*_0_. Therefore, *fz*^−^ and *Vang*^−^ mutations are recapitulated by small *f*_0_ and *g*_0_, respectively. Furthermore, Fat/Dachsous pathway is speculated to provide global cues, thus *ft*^−^ and/or *ds*^−^ are commonly interpreted as the absence of gradient cues; we use *ds*^−^ in Fig (6) and S7 Fig.

As discussed in Model, the roles of Dsh and Pk are lumped in the cooperative interactions. We heuristically seek phenotypic similarities between the patterns induced by the knockdown of cytoplasmic proteins, and those generated by perturbing (*α*, *β*), and λ. These parameters appear in different capacities in our model; the phenotypes associated with (*α*, *β*)-mutants are distinguishable from those of λ-mutants. Surprisingly, one resembles the *dsh*^−^ phenotypes, whereas the other exhibits similarities to double mutants *pk*^−^ *dsh*^−^. These phenotypic similarities are utilized to infer the dominant roles of Pk and Dsh.

We shall clarify here that both Dsh and Pk are believed to take parts in mediating cytoplasmic interactions, thus presumably affect (*α*, *β*) and λ in a complicated fashion that depends, among other factors, on their concentrations, binding affinities, diffusion constants, and mutual interactions. Therefore their impacts on the model parameters are not easily separable. Nevertheless, we see that the *in silico* mutants bear a resemblance to the *in vivo* phenotypes, which is suggestive of a correspondence between the cytoplasmic proteins and their contributions to the model parameters representing the cooperative interactions. Below we interpret the observations, and put forward a few testable hypotheses regarding the role of cytoplasmic proteins.

### Comparison of the *in vivo* and *in silico* mutants

Results of simulations and the predicted phenotypes for all classes of mutants are shown in S7 Fig, and their schematics are tabulated in Fig (6). In all cases, except for the bottom right panel, the global cues are absent, hence *ds*^−^. The first two rows in Fig (6) belong to type-I mutations, namely under- or over-expression of membrane proteins. The first row illustrate the non-autonomy induced by mutant clones embedded in otherwise wild-type backgrounds, whereas the second row shows them in mutant backgrounds lacking one of the membrane proteins. The third row demonstrates type II in the bottom left, and type III in the bottom right panels. The thickness and the length of an arrow represent the strength and spatial extension of the non-autonomous effects, respectively. Except in the type-III mutants (bottom right panel), the red arrows should be interpreted as the *deviations* of the dipoles *relative* to the wild-type polarity; not the actual orientations of the resulting dipoles. In type-III mutants, the arrows indicate the polarity patterns in clones with enhanced geometric irregularities.

The nearly perfect agreement with the *in vivo* phenotypes involving membrane proteins, confirms that these components are incorporated appropriately in the model. In the case of cytoplasmic proteins, although experimental observations suggest minimal non-autonomy of *dsh*^−^ and *pk*^−^ clones in wild-type backgrounds [13], the autonomous effects of the two are discernible. While *dsh*^−^ clones are almost unpolarized, *pk*^−^ clones seem to remain polarized parallel to the wild-type background [13]. Contrasting the *in vivo* phenotypes with our results suggests that the mutants generated by diminished strength of cooperative interactions (*α*, *β*) are reminiscent of *dsh*^−^ clones, with minute cell polarities and almost zero non-autonomy; see Fig (6) and S7 Fig. The λ-mutants, on the other hand, resemble the *pk*^−^ clones to some extent, though with imperfect alignment within the clone unlike their putative *in vivo* counterparts; indeed they look more like *dsh*^−^ *pk*^−^ double mutants. Therefore, we hypothesize that while both Dsh and Pk contribute to the magnitude and the length scale of nonlocal cytoplasmic interactions, Dsh is mainly in charge of the magnitude, whereas the length scale depends on Pk as well as Dsh. This hypothesis leads to the following important prediction. We recall from Results that the minimum concentration of Vang required for tissue polarization increases as the magnitudes of cooperative interactions (*α*, *β*) decrease; see Sec. (2.1) in S1 Appendix. Therefore we predict that under-expression of Vang can be partially compensated for, by over-expression of cytoplasmic proteins, mainly Dsh. This is an elegant manifestation of the collaboration between cytoplasmic and membrane proteins in establishing long-range polarization. Also comparing the phenotypes of membrane proteins with cytoplasmic ones, we notice that the non-autonomous effects of the former are generically stronger than those of the latter; see S7 Fig. This is consistent with the fact that cell-cell communications occur through membrane proteins, whereas cytoplasmic proteins are the carriers of intracellular interactions.

We discussed previously in Introduction that local geometric disorders were observed in Ref. [20], to obstruct the propagation of wild-type polarization. In *Drosophila*, *fat*^−^ mutants not only lack global cues, but also exhibit geometric irregularities. In order to separate the two effects of *fat*^−^, local disorder is induced by *PTEN*^−^, which does not interfere with the global cue. Ma, et.al. showed in Ref. [20], that while the alignment of polarization field is preserved in single mutants *fat*^−^ or *PTEN*^−^, the angular correlation is disrupted significantly in double mutants *fat*^−^ *PTEN*^−^, implying that local geometric disorder is an obstacle to the faithful propagation of polarization. In order to test our model’s predictions in this class of mutants, we simulate type-III mutants in the absence, as well as in the presence of global gradient cues. In our simulations we borrowed from Ref. [20], the statistics of cells’ areas in *PTEN*^−^ clones, and introduced a patch with strong geometric disorder and shrunken cells; the disorder dissolves smoothly into an otherwise wild-type background with small disorder. In the absence of graded global cues, the polarity field shows strong aberrations with swirling patterns centered at the mutant patch. Adding the global cue, however, unwinds the swirls and the wild-type polarization reappears; see the two bottom right panels in Fig (6), and S7 Fig. Disrupted polarity in cells with altered geometry can be understood in our model, by noting that nonlocal interactions sustain polarity only within a certain range of λ/*ℓ*_0_. Upon decreasing the cell size, this ratio exceeds the upper bound of the functional range of NLCI, thus the polarization is distorted. We recall that the loss of functionality of the nonlocal interactions in small cells, originates from the destabilizing competition between upregulating and downregulating interactions that strongly couple bound complexes on the opposite sides of the cells.

Comparison with experimental observations [13, 21, 31] (type I), and [13, 27, 28] (type II), and [20] (type III) reveals qualitative similarities between the *in vivo*, and *in silico* phenotypes. Agreement with experimental observations lends support to the capabilities of our model in explaining and capturing the salient features of different PCP components, and their coupling with global cues as well as geometry. Note that all of the phenotypes discussed above, ensue from a dysfunctional NLCI mechanism of some sort, and would not appear otherwise. It is also noteworthy that while the phenotypes of different, say *fz*^−^ alleles, are qualitatively similar, the spatial extension and magnitude of non-autonomy varies among them. For instance, autonomous *fz* alleles have phenotypes similar to *dsh* clones. Based on this observation, Amonlirdviman, et.al hypothesized in Ref. [13] that while these alleles might be deficient in complexing with Dsh, their ability to complex with Vang remains unchanged. Our model does not accommodate such minor effects, and is only meant to capture the primary roles of distinct PCP components.

## Discussion

In this paper we studied the role of cytoplasmic interactions in PCP through a generalized reaction-diffusion model equipped with nonlocal intracellular interactions. Although we relied on details pertaining to the core PCP pathway to interpret the results, the structure and elements of the model remain independent of pathway-specific assumptions regarding the molecular details and interactions. Thus, we believe as long as the reaction-diffusion-type mechanisms dominate the cytoplasmic transport of proteins, our model would be capable of explaining—at least qualitatively—the behavior of tissue polarity. We explored different scenarios for intra- and intercellular interactions, and specified the optimal range of cytoplasmic interactions length scales to achieve long-range polarization: 0.2 ≲ λ/*ℓ*_0_ ≲ 0.7. Investigating the cases of unequal λ_*u*_ and λ_*d*_, in particular local activation—nonlocal inhibition, we demonstrated the inefficacy of the latter in preserving the angular correlation of polarity, compared to the case of identical λ’s. We further examined the response of polarization to external cues, and concluded that NLCI is essential to detecting directional signals in even moderately disordered tissues.

A direct consequence of NLCI is the readout of cellular geometry. Of particular interest to our study is tissue elongation, a putative symmetry-breaking cue. We showed that NLCI is responsible for stabilization of collective polarity perpendicular to the axis of elongation. Agreement with the observed value of elongation at which the perpendicular polarity becomes detectable, is suggestive of the NLCI as the dominant PCP mechanism in systems like mammalian cochlea and skin. We shall emphasize that this prediction is only valid under the following assumptions: (a) polarization is predominantly induced by reaction-diffusion processes, and (b) tissue rearrangements and dynamics are negligible in comparison with PCP kinetic timescales. Lastly, in order to examine the predictive power of the model, we studied three classes of mutants and found arguably similar phenotypes to the experimental observations, which helped us interpret the model parameters and predict the role of the cytoplasmic proteins.

### Manifestations of nonlocal cytoplasmic interactions

Throughout the paper we discussed the significance of nonlocal interactions in establishing and stabilizing the long-range polarization in epithelial tissues. Furthermore, we claimed that nonlocal interactions are essential to geometric readouts, an example of which is the detection of the tissue elongation. While the coupling of geometry and polarization is a corollary of nonlocal cytoplasmic interactions, it has implications beyond stabilizing the long-range polarization, that can serve as benchmarks for experimental verification of the proposed mechanism. Two possible scenarios are discussed here.

Consider a scenario where a tissue is prepared in a polarized state, e.g. through exposure to a strong gradient cue. If the tissue is now stretched *parallel* to the axis of its initial polarization, the proteins would redistribute to rotate the polarity *perpendicular* to the axis of elongation, which is the stable configuration of polarity. Another way to view this is as follows. Suppose a gradient cue is imposed perpendicular to the polarity of an already polarized tissue. If the tissue is *not* elongated (i.e. isotropic), the polarity would rotate to align with the cue. On the other hand, if the tissue is elongated, with its initial polarity perpendicular to the axis of elongation, the newly applied global cue would be parallel to the elongation axis (since perpendicular to perpendicular is parallel). Unlike in the isotropic case, the polarization in elongated tissue would show resistance against rotation and alignment with the gradient cue. Again, this is because of the stable and favorable state of polarization being perpendicular to elongation axis; were the interactions local, the polarization would be enslaved to the global cue regardless of the tissue geometry. More importantly, even if a sufficiently strong cue manages to rotate the polarization, the proteins would redistribute back on the long junctions upon removing the global cue.

Note that nonlocality—of some sort—is required for the geometric information to become available to the proteins. Therefore, verification of the geometric readout would support the hypothesis of nonlocal interactions. The geometric information stored in packing disorder seems to be experimentally inaccessible—at least in the context of PCP—as the average polarization appears to be insensitive to the packing disorder. Elongation, as a large-scale property, provides an experimental observable to validate or reject the hypothesis of nonlocal cytoplasmic interactions. Below we propose experiments to assess our model’s predictions.

### Comparison with other models

Although our model is not the first phenomenological approach to the problem of PCP, we believe that the features included in this model, captures a broader range of recently observed phenomena. Among the models that incorporate intracellular interactions, the one put forward by Burak and Shraiman in Ref. [14] is closely related to our model. They demonstrated that in *ordered* tissues, nonlocal inhibitory reactions are sufficient to fully drive the intracellular segregation of Fz and Vang. The role of geometric disorder, however, remained to be investigated. The theoretical considerations presented here address this question, as well as the importance of nonlocal activation interactions. Interestingly, we observed that the two seemingly unrelated factors that are missing in Ref. [14], become important in relation to one another, and that the nonlocal activation is the reliable mechanism to stabilize the long-range polarity—at least in disordered tissues.

Our model suffers from several limitations, most of which arise from the phenomenological nature of our approach that neglects pathway-specific details, thus fails to provide explanations for certain observations. Unlike the models in which the parameters are inferred from experiments; e.g. [12, 13, 16], our model’s ingredients are meant to capture effective quantities such as timescales, magnitude of interactions and binding affinities. This approach enables exploring the parameter space, identifying the primary roles of distinct PCP components, and making testable predictions. There is also a minor simplifying assumption associated with the uniform junctional distributions of membrane proteins. Several experiments have suggested localization of proteins in clusters at distinct loci (puncta). The primary motivations for this approximation are the significant decrease in (a) the computational cost, and (b) model complexity. Nonetheless, by segmenting each junction into pieces of length 0.1*ℓ*_0_, we performed a limited number of simulations in the cases of small and large geometric disorders. While the approximation of uniform concentrations seems to have minimal impacts on the correlations, we suspect that dropping this assumption would indeed enhance the rotational freedom of the polarization, which could slightly improve the angular correlations in disordered systems.

### Predictions

A summary of our model’s predictions is as follows. (1) By comparing the phenotypes we concluded that the concentration of Dsh plays the dominant role in the magnitude of the cooperative interactions (*α*, *β*). We also recall that the minimum *g*_0_ that guarantees long-range polarization is inversely related to *α*, *β*. Thus, the minimum concentration of Vang (i.e. *g*_0_) required for the stable polarization depends inversely on the concentration of Dsh (i.e. *α*, *β*), and polarity alignment can be partially retained in tissues with under-expression of Vang, by over-expression of Dsh. This prediction is crucial to understanding the collaboration of membrane and cytoplasmic proteins in PCP, and can be tested by tuning the expression levels of Dsh and Vang. (2) Since the nonlocality of interactions is proposed to be dependent on Pk and Dsh, the role of elongation as a global cue relies on the abundance of Pk and Dsh, thus the knockdown of *pk* and *dsh* would invalidate the guaranteed orthogonality of polarization and the elongation axis. (3) The length scale of cytoplasmic interactions are dependent on the cytoplasmic protein *concentrations*, perhaps due to the nonlinear effects on the diffusion constants. Interpreting λ to be an increasing function of Pk (and possibly Dsh), and given that the polarization is destabilized for λ/*ℓ*_0_ ≳ 0.8, our model predicts that excess Pk destroys the PCP alignment. Interestingly, this effect was observed in a study by Cho, et.al. in Ref. [51]. Since the nonlocal interactions are contingent upon the presence of Pk and Dsh, predictions (2) and (3) can be tested—in the absence of their respective global cues—by under- or over-expressions of Pk and Dsh, as the representatives of NLCI. (4) The direction of the polarity in disordered tissues is chosen by the geometry, is independent of initial distribution, and shows robustness against stochastic noise and small external cues. This can be tested experimentally, by comparing the response of polarized tissues to global cues in transverse direction, for ordered and disordered geometries, e.g. before and after the ordering in *Drosophila* wing.

Finally, in spite of several insightful findings regarding the mutual interplay of PCP and tissue mechanics [10, 11, 20], the relevant molecular and physical mechanisms remain elusive. Planar polarity and cell packing are known to mutually influence one another. On the other hand, cell packing is highly susceptible to mechanical stress. A natural direction for future studies would be to investigate the coupling between PCP proteins and stress-generating motor proteins, as well as the emergent tissue-level coordination of polarization and regulation of mechanical stress. Furthermore, given the role of microtubules in polarizing cells through biasing transport of membrane proteins, it is of great importance to understand the conditions under which this mechanism dominates the diffusive transport. Our study lays the groundwork for further investigations, by uncovering one of the scenarios through which PCP couples to cellular geometry.

### Methods

Dynamical simulations are carried out using Runge-Kutta method of 4th order, with the time steps of 10^−3^ *γ*^−1^, on lattices of size 40 × 40 cells. For each cell, starting from a randomized distribution of F and G proteins, the concentration of proteins evolve according to the RD equations. For each set of the model parameters (e.g. disorder *ϵ*_0_, interaction length scale λ), simulations are run for 500 initial conditions. Stochasticity in the RD equations, is incorporated by adding a Gaussian white noise to the RD equations. Using the assumption of the uniform distribution of membrane proteins along all junctions, the geometrical coefficients ($K_{\mu \nu }$) are computed for all pairs of junctions *μ* and *ν*, by integrating the interaction kernels along the two junctions; see Sec. (1) in S1 Appendix for the definition of $K(r-r^{\prime })$. Boundary conditions are chosen to be periodic along both axes. The global cues are modeled as constant gradients that exponentially decay in time. For each edge of a given cell, the magnitude M is proportional to the distance of the center of the edge from the centroid of the cell.

## Supporting information

S1 Appendix Supporting Information intends to (a) introduce rigorous mathematical formalism, (b) discuss secondary results and further elaborate on those presented in the Main Text, and (c) provide additional supporting evidence for the findings. S1 Appendix.
(PDF)

S1 FigPolarization in one-dimensional arrays of cells.(a) cartoon of a 1D array of cells. Complexes with similar and opposite polarities, activate and inhibit each other on the interfaces. The edge lengths are denoted by *ℓ*_*i*,*i*+1_. (b) shows the average polarizations against *G*_0_/*F*_0_, for different values of length disorder *ϵ*_0_ = 0 to 0.6. In ordered arrays, the critical value is $g_{0}^{*}≃0.23$. The plot is obtained by ensemble averaging over 1000 realizations of quenched disorder in arrays of 1000 cells. For *G*_0_/*F*_0_ = 0.3, (c) and (d) show the heatmaps of the cell polarities versus time (vertical axis), in an ordered array with a small bias, and in a highly disordered array (*ϵ*_0_ = 0.6) with a large initial bias.(TIFF)

S2 FigMean-field solutions in two dimensions.(a1) and (a2) show the trivial MF solutions with nonzero and zero net polarities, respectively. While (a1) is a stable solution, (a2) is destabilized by NLCI. (a3) is an illustration of a nontrivial MF solution where cell polarities point in random directions. (b1) and (b2) show the initial (red) and final (blue) distributions of $f_{i}^{\text{ubd}}g_{j}^{\text{ubd}}$ for randomized edges labels (*ij*), in SLCI and NLCI systems, respectively. The variance of the distributions decreases significantly over time, supporting the MF approximation.(TIFF)

S3 Fig Cytoplasmic segregation in NLCI and SLCI regimes. (a1) The average value of the vector sum of the partial polarities defined in Eq. (26), divided by the average magnitude *Q*, as a function of time for SLCI and NLCI systems. Evidently, the ratio drops to zero for NLCI, implying full segregation. (b1) The normalized standard deviation of cell polarities defined in Eq. (27). Zero standard deviation in the presence of nonlocal interactions implies that, unlike in the SLCI case, segregation is achieved in these systems.
(TIFF)

S4 Fig Swirls and crosses in tissues with under-expression of cytoplasmic and membrane proteins. Two examples of persistent defects. (a) shows a system with λ/*ℓ*_0_ = 0.1. Other parameters are fixed at the values mentioned in Table 1 of the Main Text. (b) A system with *g*_0_ = 0.25 (i.e. close to polarization threshold), and “wild-type” interaction length scale λ/*ℓ*_0_ = 0.5. Both systems exhibit swirling and crossing patterns that appear as long-lived steady patterns. We picked one ordered and one disordered tissue. However, in both cases of small λ and small *g*_0_, defects appear, more or less irrespective of the geometric disorder.
(TIFF)

S5 Fig Comparison of LA-NLI and NLA-NLI in disordered tissues. Rose plots illustrate, for different values of geometric disorder, *ϵ*_0_, the angular distributions of polarization fields in systems with equal and unequal length scales, λ_*u*_ and λ_*d*_. (a) Equal λ’s, with *ϵ*_0_ = 0.6. (b), (c) For λ_*d*_/*ℓ*_0_ = 0.5 and 0.01 ≲ λ_*u*_/*ℓ*_0_ ≲ 0.8, the angular distributions are shown for *ϵ*_0_ = 0.45 and *ϵ*_0_ = 0.6. (d) Angular distributions for λ_*u*_/*ℓ*_0_ = 0.5 and 0.01 ≲ λ_*d*_/*ℓ*_0_ ≲ 0.8, with geometric disorder *ϵ*_0_ = 0.6.
(TIFF)

S6 FigElongated tissues and mean-field solutions.(a1) Shows the elongated system with elongation axis passing thorough a vertex. Since the cooperative interactions increase with length, the long junctions get polarized before the shorter edges can absorb complexes. This case is twofold symmetric, like a 1D array of cells that is extended perpendicular to the elongation axis. (a2) and (a3) show the alternative elongation axis perpendicular to one of the edges. In these cases there are four long edges competing to absorb membrane proteins. The possible configurations are now fourfold, two of which are polarized perpendicular, and the other two are polarized parallel to the axis of elongation; they are shown in (a1) and (a2), respectively. The latter is destabilized by the nonlocal cytoplasmic interactions. (b) For different values of λ/*ℓ*_0_, the magnitude of cooperative self-interactions *α*_*s*_ is plotted as a function of *L*/*ℓ*_0_. (c) The angle between the average polarization and the axis of elongation, as a function of the average elongation index E (initial condition and geometry are held fixed). At E≃0.1, the polarization and elongation axis are almost orthogonal; |Φ_*p*_ − Φ_*e*_| ≃ 87°.(TIFF)

S7 Fig Patterns of polarity for different classes of *in silico* mutants. Illustrations of type I, II, and III mutants. The layout of the table is the same as that in the Main Text, and the red arrows show the directions of distortion with respect to the wild-type polarity. This table facilitates a more detailed comparison of the autonomous effects that were absent in Fig (6) of the Main Text. In particular note the differences between the polarities within the putative *dsh*^−^ and *pk*^−^ clones, that were induced by small (*α*, *β*) and small λ, respectively.
(TIFF)
