# Supplementary material for: The role of intracellular interactions in the collective polarization of tissues and its interplay with cellular geometry
Source: PLoS Comput Biol. 2019 Nov 26;15(11):e1007454. doi: 10.1371/journal.pcbi.1007454 (PMC6903760; doi:10.1371/journal.pcbi.1007454)
Supplement: S1 Appendix — (PDF) [file pcbi.1007454.s001.pdf]

Supporting Information
The Role of Intracellular Interactions in the Collective Polarization of Tissues and its Interplay with Cellular Geometry

Shahriar Shadkhoo[1,2] and Madhav Mani[3,4,5]

[1] *Kavli Institute for Theoretical Physics, University of California, Santa Barbara, California, USA*
[2] *Physics Department, University of California, Santa Barbara, California, USA*
[3] *Department of Engineering Sciences and Applied Mathematics, Northwestern University, Evanston, Illinois, USA*
[4] *NSF-Simons Center for Quantitative Biology, Northwestern University, Evanston, Illinois, USA*
[5] *Department of Molecular Biosciences, Northwestern University, Evanston, Illinois, USA*

Supporting Information (SI) intends to (a) introduce rigorous mathematical formalism and definitions of the quantities introduced in the Main Text, (b) discuss secondary results, and (c) provide further evidence that support the findings. For pedagogical purposes, after introducing the formalism, we start by analyzing the one-dimensional version of the model. Studying 1D systems is insightful and helps us develop intuition about the conditions required for the polarization to become the stable fixed point of the differential reaction-diffusion equations. The results we obtain for the 1D systems, although not directly applicable to the 2D case, help us explore the mean-field solutions in 2D systems. In particular we introduce trivial and non-trivial solutions in 2D, and compare the solutions in systems with the SLCI and NLCI. We fully discuss how segregation is accomplished in systems with nonlocal interactions, and illustrate the inability of local interactions in separating the membrane proteins. The irregular patterns of polarity (swirls and crosses) and their possible origins are explored. A detailed investigation of the impact of local upregulating cytoplasmic interactions (LA-NLI) on the stability of polarization is presented, which is accompanied by a table illustrating the rose plots of dipoles' angular distributions for various combinations of length scales and geometric disorder; see Fig. (E). This is followed by a discussion on the gradient cues, its mathematical definition and further comments on the results.

Elongated tissues are investigated in Sec. (3), where the nematic tensor of elongation is introduced, the eigenvalues and eigenvectors are calculated, and the expected value of angle of polarity is plotted against the magnitude of elongation in Fig. (Fc). The last section (4) includes a brief discussion, and a table similar to that in the Main Text which illustrates the results of numerical simulations for all mutations of interest; see Fig. (G).

1 Formalism of The Generalized Reaction-Diffusion Model

Like in the Main Text, we denote the polar complexes by F–G, namely F $\equiv$ Fz:Fmi and G $\equiv$ Fmi:Vang. Labeling two adjacent cells by i and j, the average concentrations of complexes F_i-G_j is denoted by u_{ij}; consistently the concentration of F_i-G_j is denoted by $v_{ij} = u_{ji}$. The junctions shared by cells i and j, are denoted by ij hereafter. The formation of complexes F_i-G_j is upregulated by the already-bound complexes of the same polarity, and downregulated by the opposite complexes, i.e. G_i-F_j. The key assumption in this model is that such intracellular interactions, mediated by diffusing cytoplasmic proteins (Dsh, Dgo, and Pk), are not strictly local, but instead diffuse to mediate the nonlocal interaction between the complexes localized on different junctions. The magnitude of interaction, which is proportional to the local concentration of the diffusing messenger protein, decreases exponentially with the distance between the two complexes. The concentration u_{ij} of F_i-G_j complexes at point $\mathbf{r}$ on the perimeter of cell i is governed by the following equation:

$$\frac{du_{ij}(\mathbf{r})}{dt} = \kappa f_i^{\text{ubd}} g_j^{\text{ubd}} \left(1 + \alpha \sum_{\{k\}_i} \int_{i\cap k} d\mathbf{r}' \mathcal{K}_d(\mathbf{r}-\mathbf{r}') u_{ik}(\mathbf{r}')\right) - \gamma u_{ij}(\mathbf{r}) \left(1 + \beta \sum_{\{k\}_i} \int_{i\cap k} d\mathbf{r}' \mathcal{K}_d(\mathbf{r}-\mathbf{r}') v_{ik}(\mathbf{r}')\right)$$
$$+ \eta_{ij}(\mathbf{r}, t) + \mathcal{M}_{ij}(\mathbf{r})\, e^{-t/\tau_m}. \qquad (1)$$

In the above equation, $\{k\}_i$ stands for the labels of all neighbors k of a given cell i, and $\int_{i\cap j} d\mathbf{r}'$ implies integration along the edge shared by cells i and j. The factors $\mathcal{K}_u(\mathbf{r}-\mathbf{r}')$ and $\mathcal{K}_d(\mathbf{r}-\mathbf{r}')$ are the kernels of up- and downregulating interactions, and γ^{-1} is the dissociation timescale of bound complexes. As mentioned in the Main Text, the kernels $\mathcal{K}(\mathbf{r})$ are assumed to be of the exponential form, which is deduced from the diffusive dynamics of the cytoplasmic proteins. The concentration of cytoplasmic protein obeys a diffusion equation with diffusion constant D and degradation time τ:

$$\frac{\partial c(\mathbf{r}, t)}{\partial t} = D\nabla^2 c(\mathbf{r}, t) - \tau^{-1} c(\mathbf{r}, t). \qquad (2)$$

Assuming the degradation of C is much faster than polarization dynamics, $\gamma\tau \ll 1$, it suffices to only consider the steady-state solutions of $c(\mathbf{r}, t)$ in Eq. (2):

$$D\nabla^2 c(\mathbf{r}) - \tau^{-1} c(\mathbf{r}) = 0 \qquad \Longrightarrow \qquad c(\mathbf{r}) = \int_{\hexagon} d\mathbf{r}' c_0(\mathbf{r}') \exp(-|\mathbf{r} - \mathbf{r}'|/\lambda). \tag{3}$$

Here, $\mathbf{r}$ corresponds to the specific point on the cell membrane, where the the concentration of C is the linear superposition of the concentration of proteins diffused from all around the cell; hence integration $\int_{\hexagon} d\mathbf{r}'$. Next, $\lambda = \sqrt{D\tau}$ is the diffusion length of protein C before degradation. The diffusing proteins enhance/suppress the formation of like/unlike complexes. The linear dependence of cooperative terms on the concentration $u(\mathbf{r}')$ originates from the fact that in Eq. (3), the source concentration of modified proteins, $c_0(\mathbf{r}')$, is assumed to be proportional to the local concentration $u(\mathbf{r}')$. This dependency is a function of the binding affinities of cytoplasmic proteins and the membrane-bound proteins. Altogether, the coefficients are lumped into the phenomenological constants α and β. We shall emphasize that the above derivations are merely to provide a qualitative relationship between the degradation rate, diffusion constants, and the cytoplasmic interaction length scales. As mentioned in the Main Text we are aware of the complicated dependencies of λ on the molecular details which are not captured by the above equations.

The functional forms of the interaction kernels read: $\mathcal{K}_u(\mathbf{r}) = N_u^{-1} \exp(-|\mathbf{r}|/\lambda_u)$ and $\mathcal{K}_d(\mathbf{r}) = N_d^{-1} \exp(-|\mathbf{r}|/\lambda_d)$, where λ_u, λ_d are the characteristic length scales of up- and downregulating interactions, respectively. The prefactors N_u and N_d are normalization factors, to be determined shortly. Before calculating the normalization factors, we make a detour to explain an approximation we used to reduce the computational cost of simulations.

Uniform junctional distribution of proteins. The full integro-differential equation (1) couples every pairs of points on the perimeter of a cell; solving it on a 2D array of cells is computationally expensive. A simplifying approximation is to assume uniform junctional distributions of proteins, and break the integration on the cells' perimeters down to summation over the junctions. Thus the integrations are replaced by matrix products; see Eqs. (4) and (5). For notational convenience, we introduce the following: cells are labeled by the Latin letters, $i, j, \dots$, and comprise edges labeled by Greek letters, $\mu, \nu, \dots$. For example: $i \equiv \{\mu, \nu, \dots\}$, and consistently edges, that are the cell-cell junctions, are the intersections of two sets (cells): e.g. $\mu \equiv i \cap j$. The junctional averaging is then defined as: $u_\mu = \ell_\mu^{-1} \int_\mu d\mathbf{r}\, u_\mu(\mathbf{r})$, where the integral is taken over the concentration of complex $u_\mu(\mathbf{r}) \equiv u_{ij}(\mathbf{r}) = [\mathrm{F}_i\text{– G}_j](\mathbf{r})$, along the junction μ. Thus, one can recast Eq. (1) to

$$\frac{du_\mu}{dt} = \kappa f_i^{\text{ubd}} g_j^{\text{ubd}} \left(1 + \alpha_\mu \sum_{\nu \in i} \widehat{\mathcal{K}}_u^{\mu\nu} u_\nu\right) - \gamma u_\mu \left(1 + \beta_\mu \sum_{\nu \in i} \widehat{\mathcal{K}}_d^{\mu\nu} v_\nu\right) + \eta_\mu(t) + \mathcal{M}_\mu \exp(-t/\tau_m). \tag{4}$$

In the above equation, η_μ and $\mathcal{M}_\mu$ are the noise and the global cue averaged over the length of edge μ. The cooperative interactions are reduced to matrix products of $\mathcal{K}_u$ and the vector u. Matrices $\mathcal{K}_u$ are symmetric with respect, and their elements $\widehat{\mathcal{K}}_u^{\mu\nu}$ are purely geometrical constants obtained from the following relation:

$$\widehat{\mathcal{K}}_u^{\mu\nu} = \iint_{\mu,\nu} d\mathbf{r}_\nu\, d\mathbf{r}_\mu\, \mathcal{K}_u(\mathbf{r}_\nu - \mathbf{r}_\mu). \tag{5}$$

A similar relation holds for downregulating interactions $\widehat{\mathcal{K}}_d^{\mu\nu}$. In Eq. (4), coupling constants equal $\alpha_\mu = \alpha/\ell_\mu$, $\beta_\mu = \beta/\ell_\mu$, and for the kernels we have $\widehat{\mathcal{K}}^{\mu\nu} = \widehat{\mathcal{K}}^{\nu\mu} = \iint_{\mu,\nu} d\mathbf{r} d\mathbf{r}' \mathcal{K}(\mathbf{r} - \mathbf{r}')$. The self interactions, i.e. the diagonal elements equal

$$\widehat{\mathcal{K}}^{\mu\mu} = 2\ell_\mu^2 x_\mu^{-2} \left(e^{-x_\mu} + x_\mu - 1\right), \tag{6}$$

in which $x_\mu = \ell_\mu/\lambda$. We dropped the subscripts u and d for simplicity. The effective stochastic noise on a junction μ reads: $\langle \eta_\mu(t) \eta_\mu(t') \rangle = \eta_0^2 \ell_\mu \delta(t - t')$. It is noteworthy that in reality the core proteins, for example Flamingo (Fmi) and Frizzled (Fz), are observed to be persistently localized at the puncta, the subdomains of plasma membrane; see e.g. [1].

With this background, we now calculate the normalization factors introduced above. In order to discern the net effect of interaction ranges from the effective coefficients α, β, we choose the normalization factors N_u and N_d, such that the self-interaction of an edge of length ℓ_μ equals α, namely: $\alpha \widehat{\mathcal{K}}_u^{\mu\mu} = \alpha$, or $\widehat{\mathcal{K}}_u^{\mu\mu} = 1$. The same relation holds for β and $\widehat{\mathcal{K}}_d^{\mu\mu}$. This choice of normalization ensures that the observed behavior upon changing λ's is purely due to nonlocal edge-edge coupling, not the effective coefficients of local interactions α, β. Satisfying this condition for all edges simultaneously is not possible, except for ordered tissues. The normalization constant is thus calculated for an edge with the average length of all edges, $\ell_0 \equiv \overline{\ell}_\mu$. Using the definition of kernels for an edge of length ℓ_0 from Eq. (5), we obtain:

$$N(\lambda) = \iint d\mathbf{r} d\mathbf{r}' \exp(-|\mathbf{r}' - \mathbf{r}|/\lambda) = 2\lambda^2 \left(e^{-\ell_0 \lambda^{-1}} + \ell_0 \lambda^{-1} - 1\right). \tag{7}$$

Here, both $\mathbf{r}$ and $\mathbf{r}'$ lie on a single edge with length ℓ_0.

Limit of strictly local cytoplasmic interactions (SLCI). In the limit of small $\lambda/\ell_\mu \to 0$, we get, $\widehat{\mathcal{K}}^{\mu\nu} = \delta_{\mu\nu}$, where $\delta_{\mu\nu}$ is the Kronecker delta. Thus in the SLCI limit, the equations read,

$$\frac{du_\mu}{dt} = \kappa f_i^{\text{ubd}} g_j^{\text{ubd}}(1 + \alpha u_\mu) - \gamma u_\mu(1 + \beta v_\mu) + \eta_\mu(t). \tag{8}$$

Junctional and cellular polarity. Planar cell polarity can be defined at the junctional and the cellular levels. For individual junctions, polarity is defined as the difference between the concentrations $u_{ij} = [\mathrm{F}_i\!-\ \mathrm{G}_j]$ and the opposite dimer, u_{ji}, namely $p_{ij} = u_{ij} - u_{ji} = u_{ij} - v_{ij}$. Cell polarity, on the other hand, is referred to as the asymmetric (anisotropic) distribution of membrane proteins on the cell perimeter, thus requires integration on the cell-cell junctions.

Depending on the pathway of interest, polarity can be defined either as a vector (vectorial polarity), or a nematic (axial polarity), corresponding to heterodimer and homodimer clusters, respectively. The former is a vector identified by an angle $\in [0, 2\pi)$, whereas the latter is a traceless tensor with the angle in the $\in [0, \pi)$ range. In our case of study, the polarization involves two distinct complexes, Fz:Fmi and Fmi:Vang, hence vectorial polarity. Below, we introduce both definitions.

(i) Vectorial Polarization: The polarization vector (also called dipole) associated with cell i is defined as:

$$\mathbf{P}_i = \frac{1}{2}\int_{\hexagon_i} d\mathbf{r}'\, \frac{\mathbf{r}' - \mathbf{R}_i}{|\mathbf{r}' - \mathbf{R}_i|}\, \big(u_i(\mathbf{r}') - v_i(\mathbf{r}')\big) = p_i^x \hat{x} + p_i^y \hat{y}. \tag{9}$$

Here $\mathbf{R}_i$ is a reference point within cell i, with respect to which the polarization is defined. We take this to be the geometrical center of mass of each cell. The magnitude of the cell polarity $|\mathbf{P}_i|$, and the angle $\phi_i^p \in [0, 2\pi)$ measured from the x-axis, can be computed using the following relations:

$$P_i = |\mathbf{P}_i| = \sqrt{(p_i^x)^2 + (p_i^y)^2}, \qquad \text{and} \qquad \phi_i^p = \tan^{-1}(p_i^y/p_i^x). \tag{10}$$

The polarization of the tissue with N_c cells, and its global order are characterized by the following quantities: (1) magnitude of average polarization, (2) average magnitude of polarization, and (3) the ratio of (1) and (2):

$$(1)\ :\ \overline{P} = |\overline{\mathbf{P}}| = \left|\frac{1}{N_c}\sum_{i=1}^{N_c}\mathbf{P}_i\right|, \qquad (2)\ :\ \overline{Q} = \frac{1}{N_c}\sum_{i=1}^{N_c} P_i\,, \qquad (3)\ :\ \mathcal{O} = \overline{P}/\overline{Q}. \tag{11}$$

(ii) Axial Polarization: The second definition is a measure for cell polarity when dealing with homodimers like Fmi-Fmi complexes, and determines the *axis* of polarization:

$$\widehat{\mathsf{P}}_i = \begin{pmatrix} \mathcal{P}_{i,1} & \mathcal{P}_{i,2} \\ \mathcal{P}_{i,2} & -\mathcal{P}_{i,1} \end{pmatrix} \qquad \text{where,} \qquad \mathcal{P}_{i,1} = \int_0^{2\pi} d\phi\; u_i(\mathbf{r})\,\cos(2\phi), \qquad \mathcal{P}_{i,2} = \int_0^{2\pi} d\phi\; u_i(\mathbf{r})\,\sin(2\phi). \tag{12}$$

In the above equation, ϕ is the polar angle of point $\mathbf{r}$ on the perimeter of cell i and is measured with respect to a reference axis. The magnitude of polarization equals $\mathcal{P}_i = (\mathcal{P}_{i,1}^2 + \mathcal{P}_{i,2}^2)^{1/2}$. Its orientation is determined by the angle ϕ_i^p that satisfies $\cos(2\phi_i^p) = \mathcal{P}_{i,1}/\mathcal{P}_i$ and $\sin(2\phi_i^p) = \mathcal{P}_{i,2}/\mathcal{P}_i$. In Sec. (3), we use the same formalism for the elongation tensor.

Correlation function and correlation length. Correlation function is a measure of alignment of polarization field. In order to investigate the temporal behavior of the spatial extension of alignment, we define the equal-time correlation functions. Consider an arbitrary cell at $\mathbf{R}_i$, and a vector $\mathbf{r}$ connecting it to another cell at $\mathbf{R}_j = \mathbf{R}_i + \mathbf{r}$. With no further assumption we can define a correlation function, that is dependent on the distance $r = |\mathbf{r}|$ and the relative angle of $\mathbf{r}$ and $\mathbf{P}(\mathbf{R}_i)$; we call it $\theta_{\mathbf{r},\mathbf{p}}$. The latter appears due to the vectorial nature of polarization field; there is no *a priori* reason for dipoles to be correlated equally in all directions. For a tissue of N_c cells, the correlation function at time t reads,

$$S(r, \theta_{\mathbf{r},\mathbf{p}}\,; t) = N_c^{-1}\sum_i \mathbf{P}(\mathbf{R}_i\,; t)\cdot\mathbf{P}(\mathbf{R}_i + \mathbf{r}\,; t). \tag{13}$$

The above quantity calculates at each timepoint t, the average (over all cells i) of the conditional probability that the dipole at point $\mathbf{R}_j = \mathbf{R}_i + \mathbf{r}$ acquires the value and direction of $\mathbf{P}(\mathbf{R}_j)$, given the dipole at point $\mathbf{R}_i$ equals $\mathbf{P}(\mathbf{R}_i)$. For $\theta_{\mathbf{r},\mathbf{p}} = 0, \pi$, and $\theta_{\mathbf{r},\mathbf{p}} = \pm\pi/2$, we get longitudinal and transverse correlations, respectively. In spite of this angular dependence, averaging the above correlation function over $\theta_{\mathbf{r},\mathbf{p}} \in [0, 2\pi)$, returns a weighted average of correlation as a function of $r = |\mathbf{r}|$, which is bounded by the longitudinal and transverse correlations from above and below, respectively. In spite of the distinction between longitudinal and transverse correlation lengths, the qualitative behavior of the correlation length could be captured by the angular average of the correlation function. Further discussion in this regard can be found below in SI. Sec. (2.3). Thus, we define radial correlation function:

$$S(r; t) = N_c^{-1}\sum_i \int_0^{2\pi} \frac{d\theta_{\mathbf{r},\mathbf{p}}}{2\pi}\, \mathbf{P}(\mathbf{R}_i\,; t)\cdot\mathbf{P}(\mathbf{R}_i + \mathbf{r}\,; t). \tag{14}$$

Correlation length can be obtained from the above equation:

$$\xi(t) = \frac{\int_0^{R_c} dr\, r\, S(r;t)}{\int_0^{R_c} dr\, S(r;t)}. \tag{15}$$

Here, $R_c = 40$ (cell diameter) is the largest distance between two given cells in our simulations. A perfectly correlated polarization field, is identified by $\xi = R_c/2$. The time-dependent correlation lengths for three important cases of study are shown in the Main Text Fig. (3c2).

Geometric disorder. In 1D systems, the edge lengths are $\ell_i = \ell_0(1+\epsilon_i)$, where $\epsilon_i \in [-\epsilon_0, +\epsilon_0]$ with uniform distribution, and $\langle \epsilon_i \epsilon_j \rangle = \epsilon_0^2 \delta_{ij}/6$. In 2D the level of quenched disorder is controlled by randomizing the sites of a triangular lattice, based on which a polygonal lattice is generated using Voronoi tessellation. Suppose the nodes of a triangular lattice are perturbed: $\mathbf{R}_i = \mathbf{R}_i^0(1 + \mathbf{\Delta}_i)$, with $\{\mathbf{R}_i^0\}$ the loci of the nodes in the ordered triangular lattice. The magnitude of the disorder term $\mathbf{\Delta}_i$ is uniformly distributed in range $[-\Delta_0, +\Delta_0]$, with local correlations: $\langle \mathbf{\Delta}_i \cdot \mathbf{\Delta}_j \rangle = \Delta_0^2 \delta_{ij}/3$, where δ_{ij} is the Kronecker delta function. For $\Delta_0 = 0$, the corresponding Voronoi lattice would be an ordered hexagonal lattice, hence $\epsilon_0 = 0$. By randomly displacing the nodes of the triangular lattice, we can distort the resultant Voronoi lattice. The edge-length disorder of the Voronoi lattice ϵ_i, and density of defects n_d are obtained by ensemble averaging over 10000 realizations of disordered triangular lattices of size 50×50. Defects appear for disorder $\Delta_d \gtrsim 0.25$, in the triangular lattice.

Physical considerations. Given the quantitative approach of this study, we find it crucial to clarify the term "long-range", used frequently throughout the paper. The Mermin-Wagner theorem states that "true long-range" ordering is prohibited in 2D systems with continuous (e.g. rotational) symmetries, except at zero stochastic noise. The long-range order is referred to as the algebraic decay of correlation functions with distance. The magnitude of noise in our system drops as $1/\sqrt{N_{\text{mol.}}}$, with $N_{\text{mol.}}$ the number of molecules participating in binding/unbinding reactions. Thus in the limit $N_{\text{mol.}} \to \infty$, long-range order is achieved. For finite $N_{\text{mol.}}$, a state of quasi-long range order can potentially exist.

2 Mean-Field and Numerical Solutions

Mean-field (MF) solutions are obtained by assuming that for all junctions, e.g. shared by adjacent cells i and j, the products $f_i^{\text{ubd}} g_j^{\text{ubd}}$ and $f_j^{\text{ubd}} g_i^{\text{ubd}}$ are uniform across the tissue. This assumption is backed up by the following numerical analysis. We compute the root-mean squared fluctuations of $f_i^{\text{ubd}} g_j^{\text{ubd}}$, for all the junctions with fully randomized labels, and compare the initial and final values. Figures (Bb1) and (Bb2) show that in 2D systems for both SLCI and NLCI mechanisms, the final distribution of $f_i^{\text{ubd}} g_j^{\text{ubd}}$ is noticeably narrower than the initial distribution. Intuitively this approximation can be justified by the fact that the linearized reaction-diffusion equations governing u and v, obey diffusion-like equations in the continuum limit (see [3] where 1D arrays were studied). Therefore, given that the total concentrations f_0 and g_0 are uniform across the tissue, the free proteins too, spread diffusively into a rather uniform state. Here we first elaborate on the MF solutions in 1D, then discuss the 2D case.

2.1 Mean-Field Solutions in 1D

In one dimension, cells are juxtaposed in an array and are separated by junctions. The proteins localize on both sides of these junctions and form dimers. A schematic of one-dimensional arrays can be seen in Fig. (Aa). The reaction-diffusion (RD) equations governing u and v complexes on a junction shared by cells i and $i+1$ read:

$$\frac{du_{i,i+1}}{dt} = \kappa f_i^{\text{ubd}} g_{i+1}^{\text{ubd}} (1 + \alpha u_{i,i+1}) - \gamma u_{i,i+1}(1 + \beta u_{i+1,i}). \tag{16}$$

A similar equation exists for the opposite complex $v_{i,i+1} = u_{i+1,i}$. The general RD equations introduced in Eq. (1), reduce, in 1D with strictly local interactions, to the above equation that was originally proposed by Mani, et.al. in Ref. [3], where the properties of the solutions to Eq. (16) in 1D systems are investigated elaborately. Here we briefly review the main results and show that in the steady state, the mean field (MF) solutions of ordered systems exhibit a bifurcation at a critical value of the control parameter g_0/f_0, i.e. the ratio of total amounts of proteins F and G. We start by deriving equations for $p_{i,i+1} = u_{i,i+1} - v_{i,i+1}$ and $s_{i,i+1} = u_{i,i+1} + v_{i,i+1}$, by adding and subtracting Eq. (16) and its counterpart for the opposite complex:

$$\frac{dp_{i,i+1}}{dt} = \kappa(f_i^{\text{ubd}} g_{i+1}^{\text{ubd}} - f_{i+1}^{\text{ubd}} g_i^{\text{ubd}}) + \kappa\alpha(f_i^{\text{ubd}} g_{i+1}^{\text{ubd}} u_{i,i+1} - f_{i+1}^{\text{ubd}} g_i^{\text{ubd}} u_{i+1,i}) - \gamma(u_{i,i+1} - u_{i+1,i}), \tag{17a}$$

$$\frac{ds_{i,i+1}}{dt} = \kappa(f_i^{\text{ubd}} g_{i+1}^{\text{ubd}} + f_{i+1}^{\text{ubd}} g_i^{\text{ubd}}) + \kappa\alpha(f_i^{\text{ubd}} g_{i+1}^{\text{ubd}} u_{i,i+1} + f_{i+1}^{\text{ubd}} g_i^{\text{ubd}} u_{i+1,i}) - \gamma(u_{i,i+1} + u_{i+1,i}) - 2\gamma\beta - u_{i,i+1} u_{i+1,i}. \tag{17b}$$

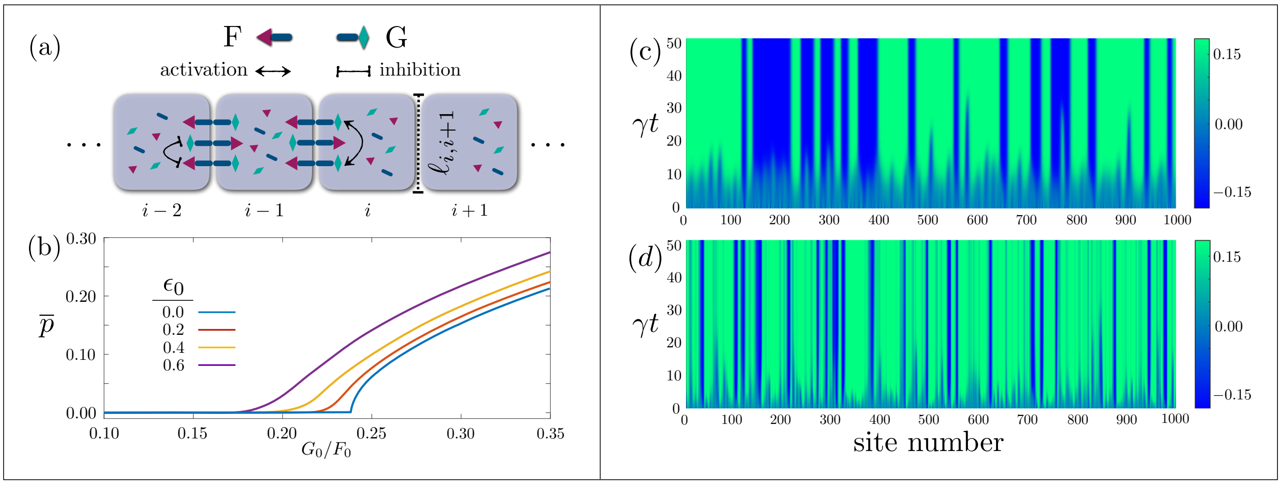

Figure A: Polarization in one-dimensional arrays of cells. (a) cartoon of a 1D array of cells. Complexes with similar and opposite polarities, activate and inhibit each other on the interfaces. The edge lengths are denoted by $\ell_{i,i+1}$. (b) shows the average polarizations against G_0/F_0, for different values of length disorder $\epsilon_0 = 0$ to 0.6. In ordered arrays, the critical value is $g_0^* \simeq 0.23$. The plot is obtained by ensemble averaging over 1000 realizations of quenched disorder in arrays of 1000 cells. For $G_0/F_0 = 0.3$, (c) and (d) show the heatmaps of the cell polarities versus time (vertical axis), in an ordered array with a small bias, and in a highly disordered array ($\epsilon_0 = 0.6$) with a large initial bias.

In the above equations we have:

$$f_i^{\text{ubd}} = \frac{F_0 - (u_{i,i+1}\ell_{i,i+1} + u_{i,i-1}\ell_{i-1,i})}{\ell_{i,i+1} + \ell_{i-1,i}} = f_{0,i} - \frac{u_{i,i+1}\ell_{i,i+1} + u_{i,i-1}\ell_{i-1,i}}{\ell_{i,i+1} + \ell_{i-1,i}}, \tag{18}$$

in which F_0 is the total amount of protein in a cell, assumed to be uniform across the tissue, and $f_{0,i}$ is the concentration of free F available to the two junctions of cell i. A similar relation can be written for g_i^{ubd} and $g_{0,i}$. In ordered systems and within the MF approximation, all of the above variables become independent of index i. A few lines of simple algebra shows that the critical value reads:

$$p^2 = s^2 - \frac{4}{\alpha\beta} \qquad \Rightarrow \qquad s^* = \frac{2}{\sqrt{\alpha\beta}} \qquad \Rightarrow \qquad g_0^* = \frac{\gamma/\kappa\alpha}{1 - \sqrt{1/\alpha\beta}} + \sqrt{\frac{1}{\alpha\beta}}. \tag{19}$$

For the values of the parameters used in the simulations, i.e. $\alpha = \beta = 5$, and $\gamma = 1$, $\kappa = 10$, the value of g_0^* is found to be $g_0^* = 0.225$, which can also be seen in Fig. (2) of Main Text. Note that in the above equations, for $\alpha\beta \to 0$, the bifurcation point diverges, implying that cooperative interactions are essential to the emergence of collective polarization. A closer look at Eqs. (17) makes it clear that the relevant parameters in the *local* bistability of the polarization are $f_i^{\text{ubd}} g_{i+1}^{\text{ubd}}$, and $f_{i+1}^{\text{ubd}} g_i^{\text{ubd}}$. When normalized by the total concentration $f_0 = F_0/\ell_0$ as the unit of concentration, one can rewrite the above parameters in terms of $f_{0,i}g_{0,i+1}$ and $f_{0,i+1}g_{0,i}$. Therefore, for disordered systems where $f_{0,i}$ and $g_{0,i}$ are no longer uniform, the local bifurcation point is randomized and the singular transition turns into a smooth cross-over.

Numerical solutions, Fig. (Ac) and (Ad), suggest that in the limit of small stochastic noise and initial bias, the steady state is not guaranteed to be uniformly polarized. The initial imbalance of protein distributions is defined as $p_0 = |u_0 - v_0|$, with u_0, v_0, the spatial averages of initial dimers' concentrations. The bias $\delta p_0/p_0$, is the normalized magnitude of spatial fluctuations of initial polarity; small and large biases correspond to $\delta p_0/p_0 \gtrsim 1$ and $\delta p_0/p_0 \lesssim 1$. While in ordered systems, a moderate initial bias suffices to achieve a uniform polarization, the patterns of polarity in highly disordered systems exhibit robustness, and are largely determined by the local geometry (disorder) of the array. Therefore as we observe in 1D, the quenched disorder imposes undesirable solutions, impairing the faithful transduction of directional information through PCP signaling. As we will see below, the situation gets only more complicated in two dimensions.

2.2 Mean-Field Solutions in 2D

The systems in one and two dimensions show inherently different behavior. In 1D, the proteins have only two junctions to localize. This limited number of choices and the resultant predictability are absent in two dimensions. Due to the large number of possible steady states in 2D, the initial configuration, as well as stochastic and geometrical disorders, influence the final state. We show, in this section, that nonlocal cytoplasmic interactions (NLCI) destabilize the vast majority of unpolarized fixed points in favor of the polarized ones. Furthermore, we find an optimal range of the NLCI length scale that assists with establishment of long-range alignment. In the following, we present the results for a set of parameters which lies within the polarized regime: in the units of $f_0 = \gamma = \ell_0 = 1$, we set $\alpha = \beta = 5, \kappa = 10$, and $g_0 = 1$.

The numerical solutions are presented below, but first let us attempt to gain some insight using analytical MF solutions for ordered tissues. Again, MF solutions imply uniform distributions of $f_i^{\text{ubd}} g_j^{\text{ubd}}$ for all junctions $\{ij\}$, and recall that it

may be justified by noting the diffusive nature of p, s dynamics, which in turn implies the diffusive dynamics of the free membrane proteins. We checked this assumption numerically. The value of $f_i^{\text{ubd}} g_j^{\text{ubd}}$, normalized by the mean, is plotted for all edges at the initial and final states in Fig. (Bb1) and (Bb2). In steady state, the standard deviation of the distribution is nearly independent of initial condition as well as the model parameters, and remains below $\simeq 0.05$, for both SLCI and NLCI regimes. Under this assumption, the RD equations take the same form as in 1D, except the total amount of proteins Fz and Vang are shared by six junctions instead of two. For ordered tissues, by virtue of sixfold symmetry, all junctions equally absorb the proteins, namely p, s are the same on all edges. Thus three edges would carry inward, and the other three carry outward dipoles.

Trivial mean-field solutions. Trivial mean-field solutions, in addition to the MF assumption discussed above, enjoy translational invariance of polarization along each of the three axes separately. The trivial MF solutions acquire two distinct configurations illustrated in Figs. (Ba1) and (Ba2), respectively. While the former represents a state with nonzero net polarization, the latter has zero net polarization. The solutions of the second type, are however destabilized when NLCI is included in the model. The net polarity in the polarized case can be calculated as follows: $p_c = p_e(1 - 2\cos\theta)$, where p_e is the magnitude of polarization of one edge calculated above, and θ is the angle between the two adjacent edges in ordered hexagons, which equals $p_c = 2\sqrt{s^2 - 4/\alpha\beta}$, for $\theta = 2\pi/3$.

We also consider a different situation in which edges have unequal α, β's. At this point, different values of α, and β can have various origins, that are beyond the scope of our discussion. However in the Sec. (3), we argue that unequal parameters can be a consequence of nonlocal interactions in elongated cells. Assume the three pairs of parallel edges acquire coefficients $\alpha_{1,2,3}$ and $\beta_{1,2,3}$. We consider two scenarios: (i) $\alpha_1 > \alpha_2 = \alpha_3$, and (ii) $\alpha_1 = \alpha_2 > \alpha_3$. From the results we found in the case of sixfold symmetric lattices, the onset of bifurcation is inversely proportion to $s^* \sim 1/\sqrt{\alpha}$. Thus in the first scenario, axis 1 is the first one to show instability upon increasing g_0 above g_0^*. Therefore, we have $f^{\text{ubd}} g^{\text{ubd}} = \gamma/(k\alpha_1)$, where,

$$f^{\text{ubd}} = f_0 - \frac{s_1\ell_1 + s_2\ell_2 + s_3\ell_3}{2(\ell_1 + \ell_2 + \ell_3)}, \tag{20}$$

and similarly for g^{ubd}. For the unpolarized edges we have:

$$s_{2,3}^2 + \frac{2}{\gamma\beta_{2,3}}\left(\gamma - \kappa f^{\text{ubd}} g^{\text{ubd}} \alpha_{2,3}\right) s_{2,3} - 2\kappa f^{\text{ubd}} g^{\text{ubd}} = 0. \tag{21}$$

Using $k f^{\text{ubd}} g^{\text{ubd}} = \gamma/\alpha_1$, we get:

$$s_{2,3} = \frac{1}{\beta_{2,3}}\left(1 - \frac{\alpha_{2,3}}{\alpha_1}\right) + \sqrt{\frac{1}{\beta_{2,3}^2}\left(1 - \frac{\alpha_{2,3}}{\alpha_1}\right)^2 + \frac{2\gamma}{\alpha_1}}. \tag{22}$$

Defining f_0^{eff} and g_0^{eff},

$$f_0^{\text{eff}} = f_0 - \frac{s_2\ell_2 + s_3\ell_3}{2(\ell_1 + \ell_2 + \ell_3)}, \quad \text{thus:} \quad f^{\text{ubd}} = f_0^{\text{eff}} - \frac{s_1\ell_1}{2(\ell_1 + \ell_2 + \ell_3)}, \tag{23}$$

and similar expressions for g_0^{eff} and g^{ubd}, we get for p_1, s_1:

$$p_1^2 = s_1^2 - \frac{4}{\alpha_1\beta_1}, \qquad s_1 = \left(f_0^{\text{eff}} + g_0^{\text{eff}}\right) - \sqrt{\left(f_0^{\text{eff}} - g_0^{\text{eff}}\right)^2 + \frac{4\gamma}{\kappa\alpha_1}}. \tag{24}$$

From the above analysis, we learn that in systems with unequal α's and β's, the problem essentially reduces to a one dimensional systems, with effective values for the concentrations of free proteins Fz and Vang. In the second scenario where $\alpha_1 = \alpha_2 > \alpha_3$, the third axis remains unpolarized as the other two are effectively more absorbent, and share the total bound proteins; thus are equally polarized with fourfold symmetry. As mentioned above, one situation of interest in which the coefficients α, β acquire unequal values on different edges, is the elongated tissues. In such systems, the above two cases correspond to Figs. (Fa1), (Fa2), and (Fa3), respectively; see Sec. (3).

Nontrivial mean-field solutions. The nontrivial solutions in 2D satisfy a weaker constraint; they do not possess the translational invariance of cell dipoles. The full analytic treatment of this problem is cumbersome. We briefly touch upon the solutions to provide some insight into the richness of the basin of attraction for a system in SLCI limit. The constraint of nontrivial solutions demands the junctional polarization p and the total amount of proteins s to be identical for all junctions. Thus in practice the only constraint is that three junctions are positively polarized and the other three carry negative polarizations; i.e. three outgoing and three incoming dipoles; see Fig. (Ba3). The numerous configurations satisfying this condition, outnumber the trivial solutions by a large margin. Note that the trivial solutions also satisfy the above constraint, but are very special configurations. Therefore, if the segregation is not carried out systematically across the tissue, the membrane proteins are extremely unlikely to redistribute in a polarized fashion; unless the initial distribution to begin with is nearly polarized. As mentioned previously, nonlocal cytoplasmic interactions destabilize the majority of nontrivial solutions, and drive the system towards a segregated state where the polarized trivial solution appears.

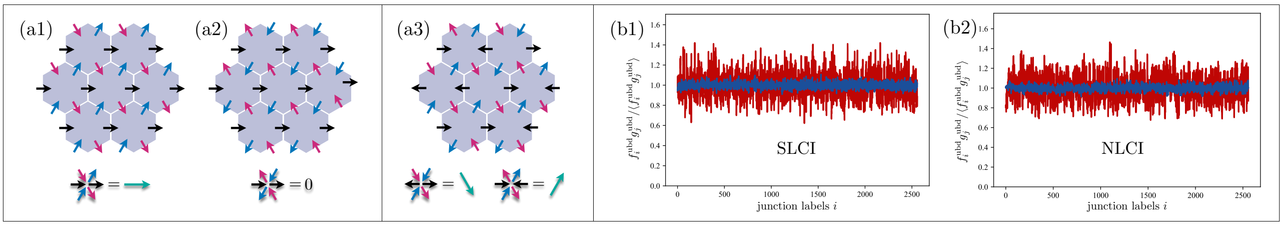

Figure B: Mean-field solutions in two dimensions. (a1) and (a2) show the trivial MF solutions with nonzero and zero net polarities, respectively. While (a1) is a stable solution, (a2) is destabilized by NLCI. (a3) is an illustration of a nontrivial MF solution where cellular polarities point in random directions. (b1) and (b2) show the initial (red) and final (blue) distributions of $f_i^{\text{ubd}} g_j^{\text{ubd}}$ for randomized edges labels (ij), in SLCI and NLCI systems, respectively. The variance of the distributions decreases significantly over time, supporting the MF approximation.

2.3 Numerical Solutions in 2D

While in relatively ordered tissues with NLCI, and in the absence of orientational cues, the polarity is determined purely by chance (stochastic noise and initial conditions), highly disordered systems show robustness against such random factors. Instead, the fixed points of polarization fields are determined collectively by the geometry of the lattice. In finite-size systems, the geometrical disorder provides a bias towards one orientation over others. This effect gets progressively more pronounced with increasing range of NLCI and/or level of the quenched disorder. External cues of sufficiently large magnitudes, however, reorient the polarity. A typical configuration of the steady states is illustrated in Main Text Fig. (3a2). We find a range of interaction length scale, $0.2 \lesssim \lambda/\ell_0 \lesssim 0.7$, for which the NLCI guarantees the long-range alignment of polarization. The directional correlation shows a peak at around $\lambda/\ell_0 \simeq 0.5$. This range of λ also depends on the geometric disorder, which hinders the establishment of polarization, and increases the lower bound of the range. For example, with $\epsilon_0 = 0.6$, the range changes to $0.25 \lesssim \lambda/\ell_0 \lesssim 0.7$. Before addressing the segregation, we note that for $\lambda/\ell_0 \gtrsim 0.7$, all edges within a cell strongly couple to each other, rendering the system fragile against stochastic noise.

Segregation mechanisms in SLCI vs. NLCI. Through a comparison of the dynamics of SLCI and NLCI in Figs. (3b1) and (3b2) of the Main Text, the role of cytoplasmic nonlocal interactions in cell-cell interactions becomes evident. While the average polarization $\overline{P}(t)$ remains negligible in SLCI limit, it saturates to the average magnitude $\overline{Q}(t)$ in systems with NLCI, which reflects the angular correlation of cell-cell polarities. More elaborately, during the first stage of dynamics, the nonlocal cytoplasmic interactions prepare each and every cell for later intercellular communications. The coarsening and propagation of polarization is then carried out by cell-cell interactions, which increase with the magnitude of cellular dipoles $Q_i(t)$. Here, an important question arises, regarding the interpretation of $\overline{Q}(t)$: Can $\overline{Q}(t)$ be thought of as a measure of cellular segregation of proteins? A naïve guess would be that since $Q_i(t)$ is oblivious to the direction of polarity, it only measures the magnitude of the dipoles per cell, which is a candidate for quantifying segregation. This, however, warrants a careful investigation, as one can see that $\overline{Q}(t)$ shows arguably similar behavior in both SLCI and NLCI cases. Does this rule out the lack of segregation in SLCI? One possibility is that while the average $\overline{Q}(t)$ increases rapidly like in the NLCI case, the segregation is not achieved consistently in all cells. This is in part due to initial condition. Consider a tissue with randomly distributed proteins on the membranes. The magnitude of a cell's dipole increases due to the localization of some of the free proteins on the membranes. Above the polarization instability, namely for large enough g_0, edges with higher initial concentration of a certain protein absorb more free protein of the same kind due to the cooperative interactions. Therefore, the final polarity depends, among other factors, on the initial condition. This is a separate effect from nonlocal interactions, and is built in the nonlinearity of RD equations regardless of the length-scale of nonlocal interactions. In order to directly measure the segregation, we define partial polarities as follows:

$$\mathbf{P}_i^F = \int_{\hexagon_i} d\mathbf{r}\, \frac{\mathbf{r} - \mathbf{R}_i}{|\mathbf{r} - \mathbf{R}_i|}\, u_i(\mathbf{r}), \tag{25}$$

and similarly $\mathbf{P}_i^G$ is obtained by substituting $v_i(\mathbf{r})$ for $u_i(\mathbf{r})$. Perfect segregation then corresponds to $\mathbf{P}_i^F = -\mathbf{P}_i^G$ in the steady state, regardless of the initial distributions. The less the level of segregation, the less the deviation from the initial condition over the time evolution: $\mathbf{P}_i^F \sim \mathbf{P}_i^F(t=0)$ and $\mathbf{P}_i^G \sim \mathbf{P}_i^G(t=0)$. Therefore, by comparing the final to initial partial polarities, one can easily signify the differences between NLCI and SLCI mechanisms, in terms of the cytoplasmic segregation. In order to quantify the segregation level, and compare that in the two mechanisms of SLCI and NLCI, we introduce the following quantities:

1. the spatial average of the magnitude of: $\mathbf{S}_i = \mathbf{P}_i^F + \mathbf{P}_i^G$. Note the vector sum, thus for perfect segregation we get: $\mathbf{S}_i = 0$. (Overlines mean spatial average over all cells at a given time).

$$\frac{\overline{S}(t)}{\overline{Q}(t)} = \frac{\overline{|\mathbf{S}_i(t)|}}{\overline{Q}(t)} = \frac{\overline{|\mathbf{P}_i^F(t) + \mathbf{P}_i^G(t)|}}{\overline{Q}(t)}. \tag{26}$$

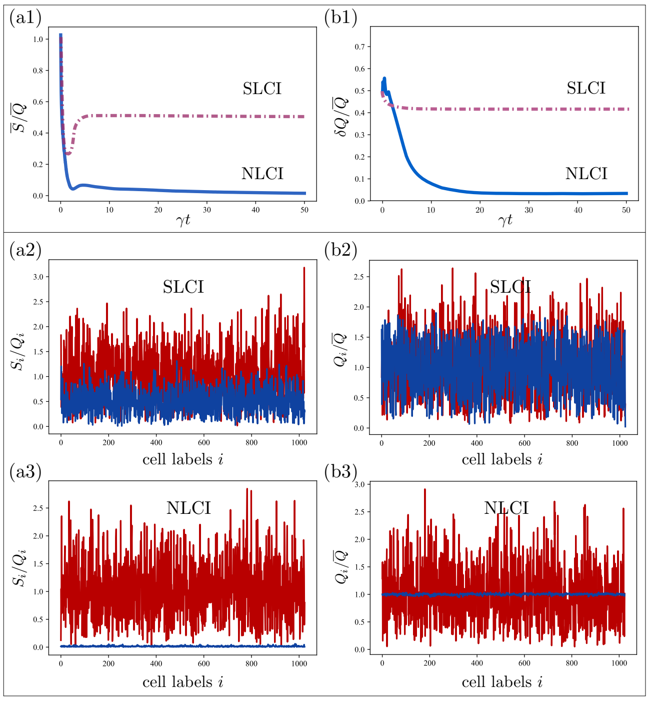

Figure C: Cytoplasmic segregation in NLCI and SLCI regimes. (a1) The average value of the vector sum of the partial polarities defined in Eq. (26), divided by the average magnitude Q, as a function of time for SLCI and NLCI systems. Evidently, the ratio drops to zero for NLCI, implying full segregation. (b1) The normalized standard deviation of cell polarities defined in Eq. (27). Zero standard deviation in the presence of nonlocal interactions implies that, unlike in the SLCI case, segregation is achieved in these systems.

2. the standard deviation of Q_i normalized by its mean,

$$\frac{\delta Q(t)}{\overline{Q}(t)} \equiv \frac{\left(\overline{|Q_i(t) - \overline{Q}(t)|^2}\right)^{1/2}}{\overline{Q}(t)}, \tag{27}$$

The former characterizes the asymmetry of protein distributions, and the latter measures the consistency in segregation among the cells. We plot the above quantities as functions of time in Figs. (Ca1) and (Cb1), respectively. In (a1), while the ratio approaches zero in steady state for the system with NLCI, it remains finite ($\simeq 0.5$) in the SLCI case, clearly showing the lack of segregation. In (b1) we see that in SLCI, the normalized standard deviation drops slightly from 0.5 at $t = 0$ to 0.4 in steady state, whereas in the NLCI case, it drops to nearly 0. The latter implies that segregation is fully achieved in all cells, and the individual cell polarities are very much close to the average polarity. Therefore, as suspected, in SLCI only the average value of $\overline{Q}$ grows, whereas cells are not coherently polarized across the tissue.

In order to show explicitly the cell-by-cell distributions of initial and final values of S_i and Q_i, and compare SLCI and NLCI cases, we plotted these quantities in Fig. (C). In (a2) and (a3), the initial (red) and final (blue) cell-by-cell distributions of S_i/Q_i are illustrated for SLCI and NLCI, respectively. Again, the nearly zero final values of the ratio for NLCI shows perfect segregation. The cellular polarity normalized by spatial average at the corresponding time-point, i.e. initial and final, are shown in (b2) and (b3). The width of the distribution shrinks dramatically in NLCI, whereas it remains comparable to its initial value in the SLCI case.

Longitudinal vs. transverse correlations. As discussed above, and according to Eqs. (14) and (15), correlation function and length are dependent on the relative angle of the reference dipole and the connecting position vector, and at least in our case is stronger in the longitudinal compared to transverse directions. Intuitively, the polarity of a given cell points towards the edges with higher concentrations of localized proteins. These edges are shared with neighbors that are located rather longitudinally with respect to the axis of polarity. The polarities of these neighbors too, are influenced by the shared edges. Therefore longitudinal correlations are stronger than the transverse correlations. This discrepancy leads to formation of transient vortex-like structures, but the steady-state behavior of the correlation length remains qualitatively the same. We ignore this effect and compute the effective correlation length by averaging over all angles.

Vortices and saddles. Several theoretical [4, 5, 6] and experimental studies [7, 8, 9] have observed swirls and saddles as different forms of the so-called "topological defects". Such defects appear either as steady or transient patterns. Steady defects can be an indication of mutations of various origins; geometric [9], or genetic [10, 11, 4, 12, 13, 14, 11]. In

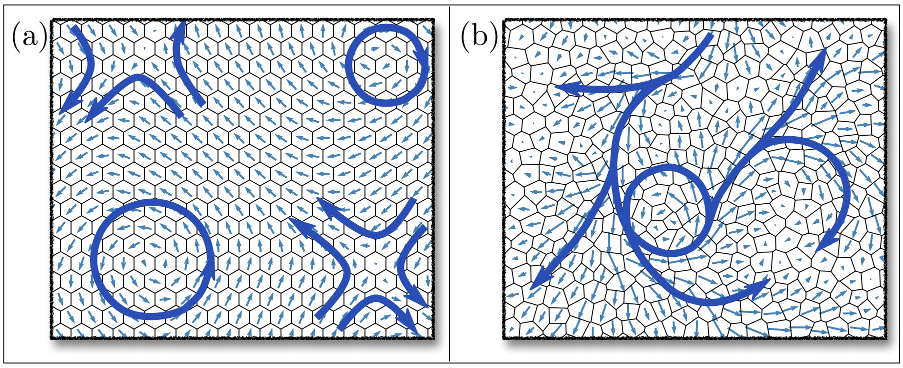

Figure D: Swirls and crosses in tissues with under-expression of cytoplasmic and membrane proteins. Two examples of persistent defects. (a) shows a system with $\lambda/\ell_0 = 0.1$. Other parameters are fixed at the values mentioned in Table (1) of the Main Text. (b) A system with $g_0 = 0.25$ (i.e. close to polarization threshold), and "wild-type" interaction length scale $\lambda/\ell_0 = 0.5$. Both systems exhibit swirling and crossing patterns that appear as long-lived steady patterns. We picked one ordered and one disordered tissue. However, in both cases of small λ and small g_0, defects appear irrespective of the geometric disorder.

Drosophila wing, where Fz and Vang localize distally and proximally, respectively, the coupling to global cues is believed to be dependent on the presence of Fat [10, 9]. While small *fat*$^-$ clones ($\lesssim$ 10 cell diameters), exhibit little deviation from wild-type polarization, swirling patterns appear in larger clones, where the polarity is aligned over clusters of (roughly) 10 cells; also implying that Fz feedback loops are left intact in *fat*$^-$ patches. Therefore, the propagation of polarization across neighboring cells is carried out through Fz feedback loop, and the global alignment is achieved through coupling to the cues. Our model predicts both transient and steady swirls, depending on the values of the model parameters. We observe two distinct types of steady defects: (a) As λ is increased from SLCI to NLCI regime, there exists a narrow range $0.05 \lesssim \lambda \lesssim 0.15$, over which vortices and saddles appear as long-lived patterns; Fig. (Da). (b) Another situation where swirls appear is when $g_0 \lesssim g_0^*$, i.e. under-expression of one of the membrane proteins, say Vang, that is interpreted as a global mutation within the context of our model; Fig. (Db). Such patterns are indeed observed in Vang mutants [7]. Local mutations of various kinds are fully discussed in Main Text and below in SI. Sec. (4).

An interesting observation regarding the second type, i.e. $g_0 \lesssim g_0^*$, is that for each specific realization of the geometrically disordered tissues, the characteristics of the steady-state pattern of polarity (e.g. positions of swirls) are left almost intact, as g_0 increases from $\simeq 0.2$ to $\simeq 0.3$. The magnitudes of dipoles, however, increase. Consequently, the swirls and branches gradually merge and align for $g_0 \gtrsim 0.3$, and long-range polarity emerges. This behavior, *in disordered tissues*, is independent of the initial condition and stochastic noise, implying that the patterns of polarization are fixed by the the cell geometry of the tissue in regimes where the correlation lengths are of the order of a few cell diameters. In general, and as briefly mentioned above, in NLCI the geometrical information is detected through nonlocal interactions. The coupling to geometry provides local biases for cell dipoles. As g_0 increases, the correlation length increases and the global direction is chosen collectively.

Equal vs. unequal length scales of cytoplasmic interactions. In the Main Text we discussed the effects of unequal nonlocal cytoplasmic interactions that appear in upregulating and downregulating kernels, i.e. λ_u and λ_d. Here we elaborate on that discussion and consider various cases to investigate the role of these length scales, and the behavior of the angular correlation of polarity. In each case the geometry of the tissue is held fixed. The geometric disorder is denoted by ϵ_0.

(1) For $\epsilon_0 = 0.5$, find the angular correlation for different values of $\lambda_u = \lambda_d = 0.01 – 0.8$.
(2) & (3) For $\epsilon_0 = 0.45$ and 0.6, with fixed $\lambda_d = 0.5$, compare the angular correlation as λ_u increases from 0.01 to 0.8.
(4) For $\epsilon_0 = 0.5$, and $\lambda_u = 0.5$, compare the angular correlation for $\lambda_d = 0.01 – 0.8$.

The findings are as follows:

(1) For equal length scales, we observe that the angular correlation increases with λ. The standard deviation of polarity is found to be smaller than 30°, for $0.2 \lesssim \lambda \lesssim 0.7$, and is maximized at around $\lambda \simeq 0.4 – 0.5$. For $\lambda \gtrsim 0.7$ the stochastic noise destroys the long-range polarization drastically. The reason is that the stabilizing and destabilizing forces compete at all points on the perimeter of each cell, namely all points are strongly coupled to one another. Thus the segregation becomes progressively more unstable as λ is increased; see Fig. (Ea).

(2),(3) For geometric disorder $\epsilon_0 \lesssim 0.45$, and for $0.2 \lesssim \lambda_d \lesssim 0.7$ there is no detectable difference between the angular correlation of systems with different values of $\lambda_u \lesssim 0.8$. Beyond this value, the correlation declines slightly, and is eventually destroyed for larger values. In order to further examine the importance λ_u in disordered tissues, we repeat the same simulations, but for larger geometric disorder $\epsilon_0 = 0.6$. Interestingly we realize that larger disorder impedes the

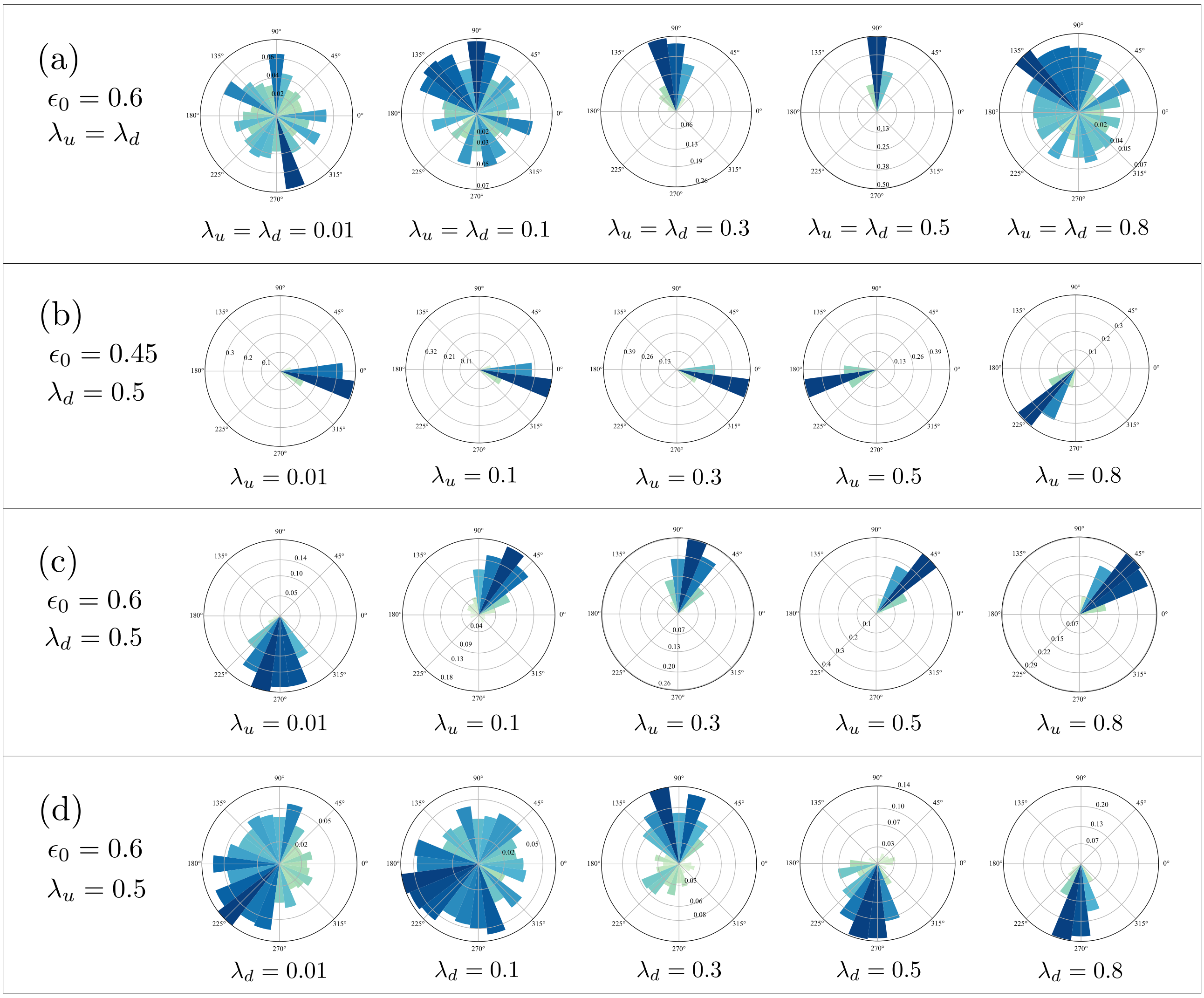

Figure E: Comparison of LA-NLI and NLA-NLI in disordered tissues. Rose plots illustrate, for different values of geometric disorder, ϵ_0, the angular distributions of polarization fields in systems with equal and unequal length scales, λ_u and λ_d. (a) Equal λ's, with $\epsilon_0 = 0.6$. (b),(c) For $\lambda_d/\ell_0 = 0.5$ and $0.01 \lesssim \lambda_u/\ell_0 \lesssim 0.8$, the angular distributions are shown for $\epsilon_0 = 0.45$ and $\epsilon_0 = 0.6$. (d) Angular distributions for $\lambda_u/\ell_0 = 0.5$ and $0.01 \lesssim \lambda_d/\ell_0 \lesssim 0.8$, with geometric disorder $\epsilon_0 = 0.6$.

long-range correlation of polarity for $\lambda_u \lesssim 0.4$. The correlated polarity is retained for $0.4 \lesssim \lambda_u \lesssim 0.7$ and is declined again for larger values; see Fig. (Eb) and (Ec).

(4) Systems with nonlocal activation and local inhibition are incapable of achieving long-range polarization. We fixed $\epsilon_0 = 0.6$ and $\lambda_u = 0.5$ and vary the inhibition length scale between $\lambda_d = 0.01 - 0.8$. The correlation grows as λ_d increases; see Fig. (Ed). An intuitive argument for why small λ_d fails to work is as follows: for $\lambda_d \gtrsim 0.8$, the unlike complexes on the opposite sides of the cell inhibit each other, e.g. Fz on the right side, inhibits Vang on the left side. Thus, the Vang proteins sitting on the left side of the same cell are destabilized by Fz from across the cell. However, as far as polarity is concerned, the Vang protein on the opposite side of the cell is indeed contributing positively to the same direction of polarity as Fz. Therefore, for $\lambda_d \gtrsim 0.8$, the polarization, even if initially correlated over long distances, becomes highly unstable against the stochastic noise. The same argument holds true for mutual stabilizing feedback of the like complexes if $\lambda_u \gtrsim 0.8$, which contributes negatively to the alignment of the polarization field.

Comparing the charts in Fig. (E), we conclude that: (1) In general geometrical disorder distorts the alignment of polarization on large length scales. (2) Equal length scales of activation and inhibition is more efficient in retaining the long-range correlation, especially in highly disordered tissues. (3) Unlike the case of equal $\lambda_u = \lambda_d$, fixing λ_u at an intermediate value, e.g. 0.5, allows for the other length scale to exceed $\lambda_d \simeq 0.7$, without losing the angular correlation. Though the correlation eventually decays for larger values, $\lambda_d \gtrsim 0.9$.

Directional cues. We consider two types of cues, bulk and boundary signals, each of which may be persistent or transient. Bulk cues couple to the F complex across the entire tissue, whereas boundary cues couple only at the boundaries. For bulk

cues in, say $+x$ direction, we use the gradient cues of constant slope in each cell: $\mathcal{M}_{ij}(\mathbf{r}) = \mathcal{M}_0\,(x_{ij} - X_i)$; where $\mathcal{M}_0$ is the slope of the cue, x_{ij} is the x-coordinate of the points on junction (ij), and X_i is that of the centroid of cell i. We simulated the response of the polarization field and observed that NLCI significantly enhances the sensitivity of the polarization field to such global cues. Before proceeding, we shall mention that there exist two time scales in this analysis: the response time scale of polarization field τ_{res}, and the persistence time scale of the cue τ_0. The results in a nutshell, are as follows: (1) Reorientation of dipoles for fixed τ_{res} and τ_0 requires weaker cues in systems with NLCI than those with SLCI. For persistent cues ($\gamma\tau_m \gtrsim 100$), NLCI responds to signals as small as $\mathcal{M}_0 \simeq 0.05$ over $\gamma\tau_{\text{res}} \simeq 2$, whereas SLCI requires at least $\mathcal{M}_0 \simeq 0.5$. The minimum $\mathcal{M}_0$ increases for smaller τ_m's. For example, in NLCI with $\mathcal{M}_0 \simeq 0.05$ a nearly persistent signal is required, whereas for larger cues $\mathcal{M}_0 \gtrsim 1$, even a rapid transient signal $\gamma\tau_m \simeq 1$ is sufficient to rotate the dipoles over the same time scale. (2) In accord with (1), and due to the small correlation length in SLCI systems, the detection of a cue in these systems happens over exceedingly larger time scales, compared to NLCI with the same magnitude. (3) In the case of boundary cues (i.e. a column of polarized cells), in nearly ordered ($\epsilon_0 \lesssim 0.2$) tissues with zero stochastic noise, SLCI suffice to detect the signal. Presence of geometric disorder and/or stochastic noise, however, necessitates NLCI for the dipoles to align with the cue. Given that the onset of PCP alignment precede the geometrical ordering of the tissue [2], NLCI seems to be the key to the detection of directional cues. Finally, an interesting observation is that, (4) NLCI systems appear to detect sufficiently large *initial* boundary signals. Initial boundary signal is implemented by polarizing a column of cells, with significantly larger asymmetry of protein distributions compared to the bulk cells. This implies that a temporary boundary signal would in principle be able to rotate the dipoles, should the cytoplasmic interactions be nonlocal.

3 Elongated Cell Geometry

Measure of elongation. Elongation is commonly parametrized using a traceless matrix $\widehat{\mathcal{E}}$. This matrix, the magnitude and the angle of elongation with respect to x-axis can be calculated as follows:

$$\widehat{\mathcal{E}}_i = \begin{pmatrix} \varepsilon_{i,1} & \varepsilon_{i,2} \\ \varepsilon_{i,2} & -\varepsilon_{i,1} \end{pmatrix}, \qquad \mathcal{E}_i = \sqrt{\varepsilon_{i,1}^2 + \varepsilon_{i,2}^2}\,, \qquad \phi_i^e = \frac{1}{2}\cos^{-1}(\varepsilon_{i,1}/\mathcal{E}_i). \tag{28}$$

For a tissue consisting of N_c cells, and with $\varepsilon_1 = N_c^{-1}\sum_{i=1}^{N_c}\varepsilon_{i,1}$, we get for the average elongation:

$$\mathcal{E} = N_c^{-1}\sum_{i=1}^{N_c}\mathcal{E}_i\,, \qquad \Phi_e = \frac{1}{2}\cos^{-1}(\varepsilon_1/\mathcal{E}). \tag{29}$$

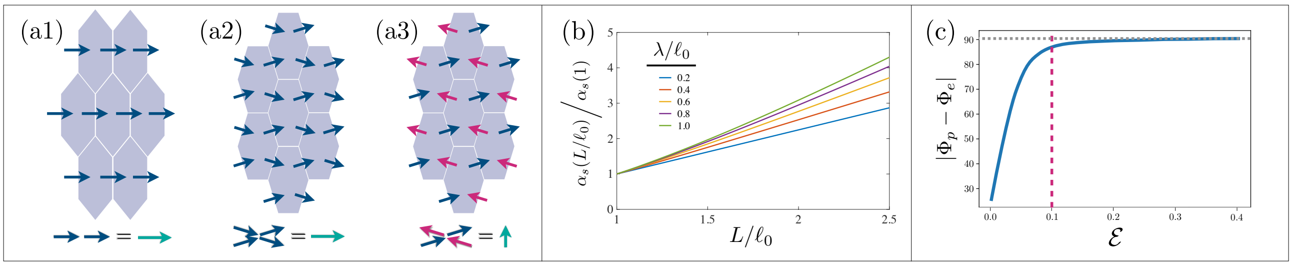

Figure F: Elongated tissues and mean-field solutions. (a1) Shows the elongated system with elongation axis passing thorough a vertex. Since the cooperative interactions increase with length, the long junctions get polarized before the shorter edges can absorb complexes. This case is twofold symmetric, like a 1D array of cells that is extended perpendicular to the elongation axis. (a2) and (a3) show the alternative elongation axis perpendicular to one of the edges. In these cases there are four long edges competing to absorb membrane proteins. The possible configurations are now fourfold, two of which are polarized perpendicular, and the other two are polarized parallel to the axis of elongation; they are shown in (a1) and (a2), respectively. The latter is destabilized by the nonlocal cytoplasmic interactions. (b) For different values of λ/ℓ_0, the magnitude of cooperative self-interactions α_s is plotted as a function of L/ℓ_0. (c) The angle between the average polarization and the axis of elongation, as a function of the average elongation index $\mathcal{E}$ (initial condition and geometry are held fixed). At $\mathcal{E} \simeq 0.1$, the polarization and elongation axis are almost orthogonal; $|\Phi_p - \Phi_e| \simeq 87°$.

In terms of the elongation index $\mathcal{E}$, we plot in Fig. (Fb), the angle between y-axis and the steady-state polarization vector $|\Phi_p - \Phi_e|$ (in degrees), versus different values of $\mathcal{E}$, for a system with the same initial condition and same *initial* lattice (i.e. before stretching), but elongated along y-axis. Here, Φ_p is defined as the angle of the axis between polarization and the x-axis, and over the domain of $\Phi_p \in [0, \pi)$. Since $\Phi_e = \pi/2$ for elongation in the y direction, we have $|\Phi_p - \Phi_e| \in [0, \pi/2]$. Of course for $\mathcal{E} = 0$, the axis of elongation is not defined, yet we measure it with respect to y-axis. It is important to note that this figure is only an example where the polarization for $\mathcal{E} = 0$, happens to make a small angle with y-axis (25°). It is clear that depending on the geometry of the lattice and the initial condition, the polarization can be perpendicular to y-axis, even for $\mathcal{E} = 0$. Therefore among different simulations, we chose one with a relatively large effect of elongation, so that the onset of perpendicular polarity becomes easily distinguishable.

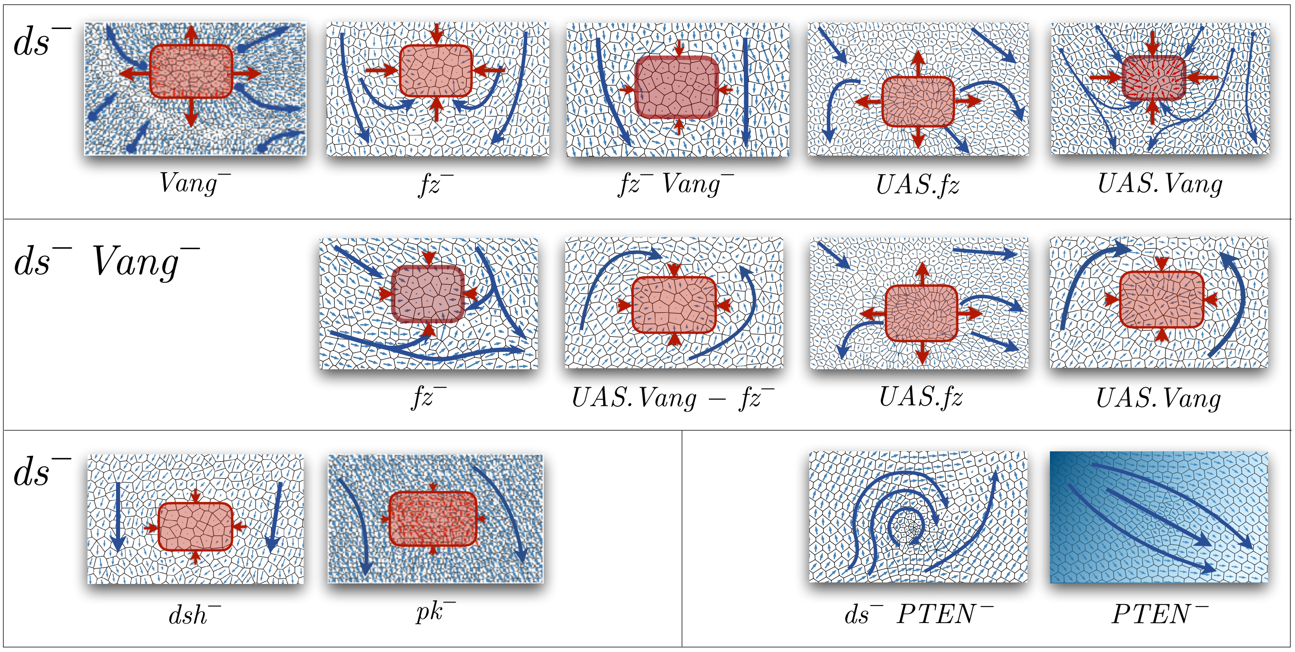

Figure G: Patterns of polarity for different classes of *in silico* mutants. Illustrations of type I, II, and III mutants. The layout of the table is the same as that in the Main Text, and the red arrows show the directions of distortion with respect to the wild-type polarity. This table facilitates a more detailed comparison of the autonomous effects that were absent in Fig. (6) of the Main Text. In particular note the differences between the polarities within the putative dsh^- and pk^- clones, that were induced by small (α, β) and small λ, respectively.

Elongation parameter $\mathcal{E}$, measures the deviation from equilateral hexagons where $\mathcal{E} = 0$. In ordered tissues, there are two possible choices for elongation axis: parallel to a pair of parallel edges, Fig. (Fa1); and perpendicular to a pair of edges; Figs. (Fa2,Fa3). While (a1) and (a2) are polarized perpendicular to the axis of elongation, (a3) exhibits parallel polarization. The latter, however, is destabilized by NLCI inhibiting localization of unlike complexes on adjacent junctions. In disordered tissues, elongation is a mixture of (a1) and (a2), both of which give rise to perpendicular polarization.

In anisotropic cells, the effective values of cooperative interactions are larger for the long junctions. Here, without deriving explicit expressions for the effective parameters in elongated system, we only argue that the longer junctions acquire larger coefficients α, β. As in the case of one dimension, searching for mean-field (MF) solutions, we assume that in the steady state the concentrations of bound proteins are translationally invariant along the three main axes of the lattice. In a self-consistent approach, the effective α, β are dependent on the concentrations of dimers on other edges. Therefore, assuming the system has reached its steady state, we can write the cooperative interactions as functions of the concentrations of proteins on all the edges, weighted by the geometrical factors originating from nonlocal interactions. For small ranges of NLCI, $\lambda \ll 0.7$, the effect of other edges are negligible and only the self-interaction of each edge is to be taken into account. Intuitively this is because the self-interaction is a monotonically increasing function of the edge length. Therefore longer edges with NLCI, have larger values of α, β. Upon increasing λ, the interactions between all pairs of edges increase. However the qualitative behavior of effective α, β's for different edges does not change. With this in mind, and using the symmetry arguments between junctions with equal lengths, one can see that the cartoons in Figs. (Fa1), and (Fa2, Fa3) correspond to, i.e. $\alpha_1 > \alpha_2, \alpha_3$ and $\alpha_1 = \alpha_2 > \alpha_3$, respectively. Using the analysis sketched in Sec. (2.2), it is easy to see that in such systems the junctions with larger α, β, are more unstable towards polarization transition (remember the critical value is inversely related to α, β). Following the above discussion on different coefficients of cooperative self-interactions α_s, and the equations derived in Sec. (2.2), we calculate these coefficients as functions of L/ℓ_0, for different λ's. Fig. (Fb) shows the numerical results for $0.2 \leq \lambda/\ell_0 \leq 1$. In order to discern the effect of the elongation from that of nonlocal interactions, we normalize all the curves by the coefficients calculated for $L = \ell_0$, such that $\alpha_s(1) = 1$ for all λ's. The coefficient of self-interaction is a monotonically increasing function of the length of junction; hence polarity emerges perpendicular to the longer junctions, as depicted in Figs. (Fa1) and (Fa2).

4 Mutants and The Corresponding Phenotypes

In the Main Text we presented the schematics of phenotypes of three types of mutants. The schematics in the Main Text Fig. (9) only show the non-autonomous effects of each clone. Here we show the actual polarization field obtained from the simulations, in which the autonomous effects within the clones are also illustrated.

Type-I Mutants: Lack of membrane proteins. Type I lacks one or both of the membrane proteins, namely f_0, g_0. The

non-autonomy is evident in all cases. In the first row of Fig. (G), non-autonomy is extended to multiple layers of cells from the clone boundaries. In the second row where the background lacks Vang as well, the non-autonomy is mostly limited to a single layer of surrounding cells.

Type-II Mutants: Lack of cytoplasmic interactions. The role of cytoplasmic proteins is deduced by comparing the *in silico* and *in vivo* phenotypes. We concluded that Dsh is mostly responsible for the local cooperative interactions with some contributions to the nonlocal part. Pk on the other hand is mainly involved in nonlocal interactions, through cytoplasmic diffusion. It can be seen in bottom left panel of Fig. (G), that in agreement with experiments, e.g. [4], the phenotypes show almost no non-autonomy. Their autonomous effects, however, are distinct. Lacking Dsh removes the cell polarity within the clone, whereas absence of Pk has only minor effects on the polarity of the clone.

Type-III Mutants: Disordered geometry. We tested the predictions of our model in the case of geometric irregularities. In Ref. [9], Ma, et.al. suggested that clones with enhanced geometric disorder—induced by under-expression of PTEN—obstruct the propagation of polarization field. This effect appears in tissues where global cues are absent. Polarity was observed to be retained when the global cue was added. As can be seen in the two right panels of the last row in Fig. (G), the system with lack of global cue shows swirling pattern of polarization. The wild-type polarity, however, reappears upon adding the gradient cue.

References

[1] Helen Strutt, Samantha J Warrington, and David Strutt. Dynamics of core planar polarity protein turnover and stable assembly into discrete membrane subdomains. *Developmental cell*, 20(4):511–525, 2011.

[2] Anne-Kathrin Classen, Kurt I Anderson, Eric Marois, and Suzanne Eaton. Hexagonal packing of drosophila wing epithelial cells by the planar cell polarity pathway. *Developmental cell*, 9(6):805–817, 2005.

[3] Madhav Mani, Sidhartha Goyal, Kenneth D Irvine, and Boris I Shraiman. Collective polarization model for gradient sensing via dachsous-fat intercellular signaling. *Proceedings of the National Academy of Sciences*, 110(51):20420–20425, 2013.

[4] Keith Amonlirdviman, Narmada A Khare, David RP Tree, Wei-Shen Chen, Jeffrey D Axelrod, and Claire J Tomlin. Mathematical modeling of planar cell polarity to understand domineering nonautonomy. *Science*, 307(5708):423–426, 2005.

[5] Yoram Burak and Boris I Shraiman. Order and stochastic dynamics in drosophila planar cell polarity. *PLoS computational biology*, 5(12):e1000628, 2009.

[6] Katie Abley, Pierre Barbier De Reuille, David Strutt, Andrew Bangham, Przemyslaw Prusinkiewicz, Athanasius FM Marée, Verônica A Grieneisen, and Enrico Coen. An intracellular partitioning-based framework for tissue cell polarity in plants and animals. *Development*, 140(10):2061–2074, 2013.

[7] Maureen Cetera, Liliya Leybova, Frank W Woo, Michael Deans, and Danelle Devenport. Planar cell polarity-dependent and independent functions in the emergence of tissue-scale hair follicle patterns. *Developmental biology*, 428(1):188–203, 2017.

[8] Wei-Shen Chen, Dragana Antic, Maja Matis, Catriona Y Logan, Michael Povelones, Graham A Anderson, Roel Nusse, and Jeffrey D Axelrod. Asymmetric homotypic interactions of the atypical cadherin flamingo mediate intercellular polarity signaling. *Cell*, 133(6):1093–1105, 2008.

[9] Dali Ma, Keith Amonlirdviman, Robin L Raffard, Alessandro Abate, Claire J Tomlin, and Jeffrey D Axelrod. Cell packing influences planar cell polarity signaling. *Proceedings of the National Academy of Sciences*, 105(48):18800–18805, 2008.

[10] Dali Ma, Chung-hui Yang, Helen McNeill, Michael A Simon, and Jeffrey D Axelrod. Fidelity in planar cell polarity signalling. *Nature*, 421(6922):543–547, 2003.

[11] Gary Struhl, José Casal, and Peter A Lawrence. Dissecting the molecular bridges that mediate the function of frizzled in planar cell polarity. *Development*, 139(19):3665–3674, 2012.

[12] David Strutt and Helen Strutt. Differential activities of the core planar polarity proteins during drosophila wing patterning. *Developmental biology*, 302(1):181–194, 2007.

[13] Samantha J Warrington, Helen Strutt, Katherine H Fisher, and David Strutt. A dual function for prickle in regulating frizzled stability during feedback-dependent amplification of planar polarity. *Current Biology*, 27(18):2784–2797, 2017.

[14] Jun Wu and Marek Mlodzik. The frizzled extracellular domain is a ligand for van gogh/stbm during nonautonomous planar cell polarity signaling. *Developmental cell*, 15(3):462–469, 2008.
