# Supplementary figures and images for: The role of intracellular interactions in the collective polarization of tissues and its interplay with cellular geometry
Source: PLoS Comput Biol. 2019 Nov 26;15(11):e1007454. doi: 10.1371/journal.pcbi.1007454 (PMC6903760; doi:10.1371/journal.pcbi.1007454)

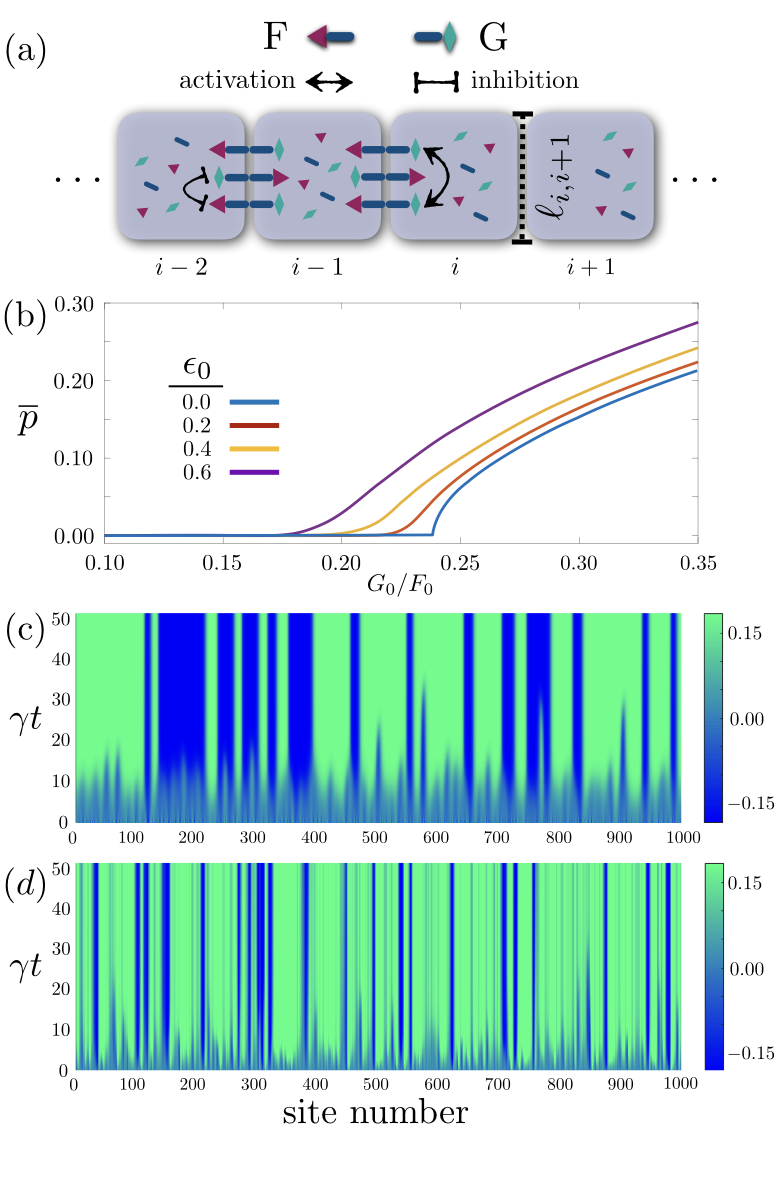

Supplement: S1 Fig — (a) cartoon of a 1D array of cells. Complexes with similar and opposite polarities, activate and inhibit each other on the interfaces. The edge lengths are denoted by ℓi,i+1. (b) shows the average polarizations against G0/F0, for different values of length disorder ϵ0 = 0 to 0.6. In ordered arrays, the critical value is g0*≃0.23. The plot is obtained by ensemble averaging over 1000 realizations of quenched disorder in arrays of 1000 cells. For G0/F0 = 0.3, (c) and (d) show the heatmaps of the cell polarities versus time (vertical axis), in an ordered array with a small bias, and in a highly disordered array (ϵ0 = 0.6) with a large initial bias. (TIFF) [file pcbi.1007454.s002.tiff]

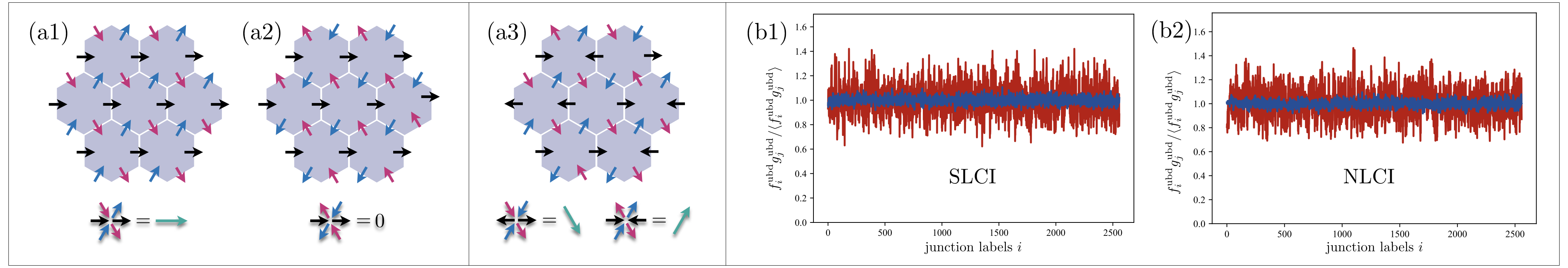

Supplement: S2 Fig — (a1) and (a2) show the trivial MF solutions with nonzero and zero net polarities, respectively. While (a1) is a stable solution, (a2) is destabilized by NLCI. (a3) is an illustration of a nontrivial MF solution where cell polarities point in random directions. (b1) and (b2) show the initial (red) and final (blue) distributions of fiubdgjubd for randomized edges labels (ij), in SLCI and NLCI systems, respectively. The variance of the distributions decreases significantly over time, supporting the MF approximation. (TIFF) [file pcbi.1007454.s003.tiff]

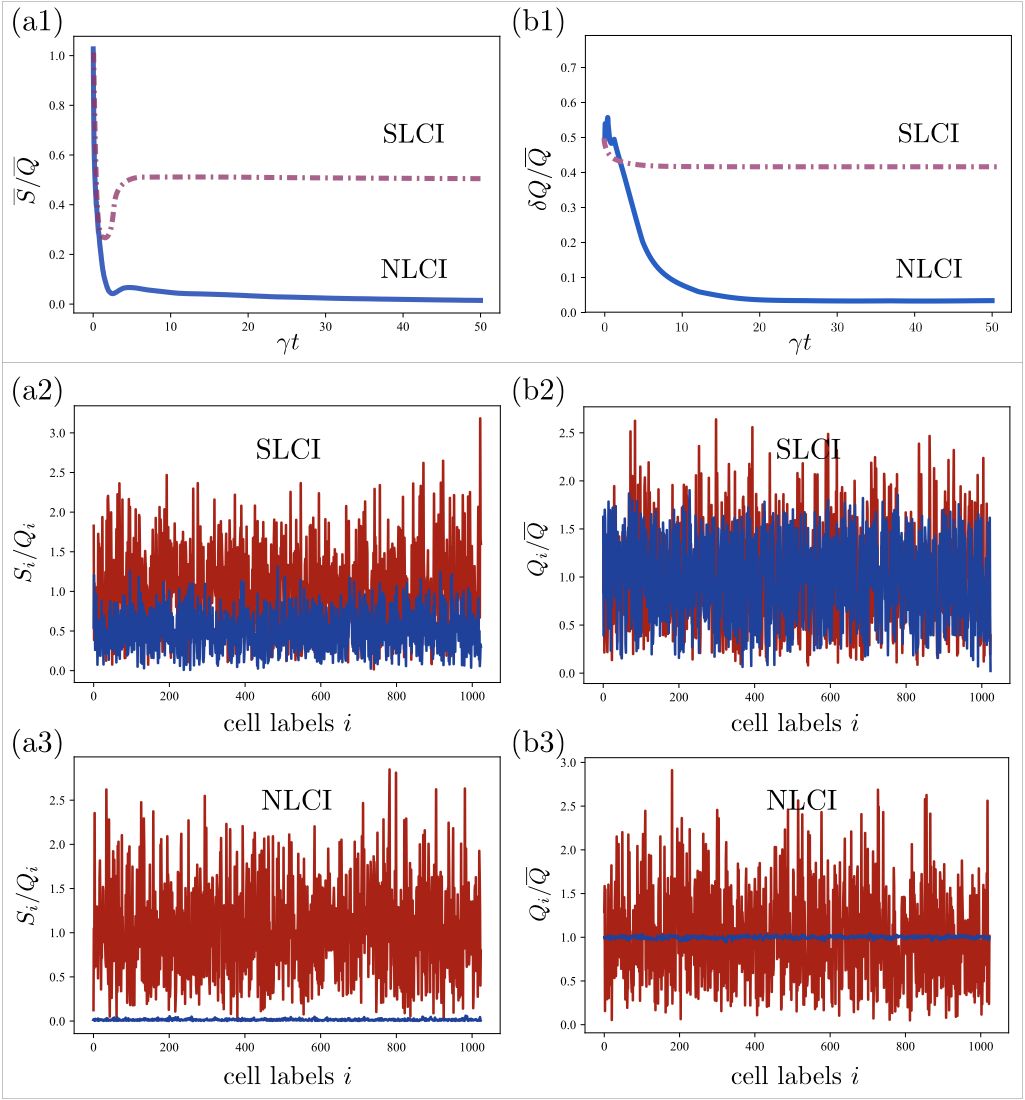

Supplement: S3 Fig — (a1) The average value of the vector sum of the partial polarities defined in Eq. (26), divided by the average magnitude Q, as a function of time for SLCI and NLCI systems. Evidently, the ratio drops to zero for NLCI, implying full segregation. (b1) The normalized standard deviation of cell polarities defined in Eq. (27). Zero standard deviation in the presence of nonlocal interactions implies that, unlike in the SLCI case, segregation is achieved in these systems. (TIFF) [file pcbi.1007454.s004.tiff]

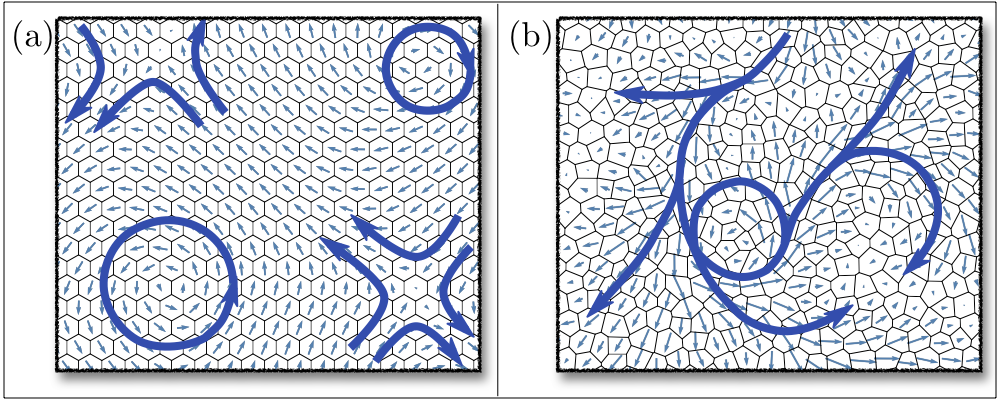

Supplement: S4 Fig — Two examples of persistent defects. (a) shows a system with λ/ℓ0 = 0.1. Other parameters are fixed at the values mentioned in Table 1 of the Main Text. (b) A system with g0 = 0.25 (i.e. close to polarization threshold), and “wild-type” interaction length scale λ/ℓ0 = 0.5. Both systems exhibit swirling and crossing patterns that appear as long-lived steady patterns. We picked one ordered and one disordered tissue. However, in both cases of small λ and small g0, defects appear, more or less irrespective of the geometric disorder. (TIFF) [file pcbi.1007454.s005.tiff]

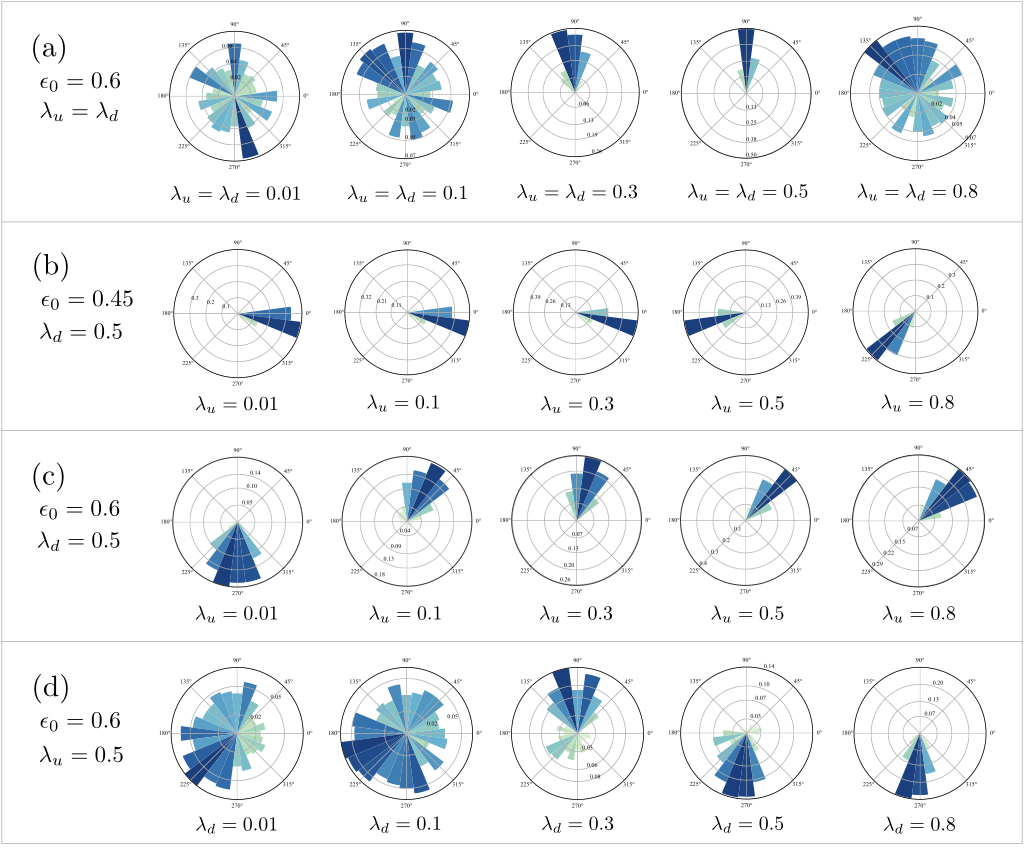

Supplement: S5 Fig — Rose plots illustrate, for different values of geometric disorder, ϵ0, the angular distributions of polarization fields in systems with equal and unequal length scales, λu and λd. (a) Equal λ’s, with ϵ0 = 0.6. (b), (c) For λd/ℓ0 = 0.5 and 0.01 ≲ λu/ℓ0 ≲ 0.8, the angular distributions are shown for ϵ0 = 0.45 and ϵ0 = 0.6. (d) Angular distributions for λu/ℓ0 = 0.5 and 0.01 ≲ λd/ℓ0 ≲ 0.8, with geometric disorder ϵ0 = 0.6. (TIFF) [file pcbi.1007454.s006.tiff]

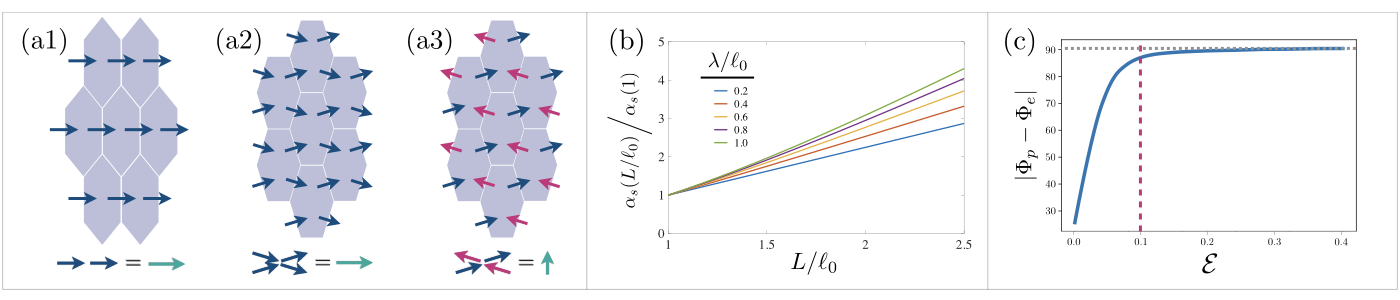

Supplement: S6 Fig — (a1) Shows the elongated system with elongation axis passing thorough a vertex. Since the cooperative interactions increase with length, the long junctions get polarized before the shorter edges can absorb complexes. This case is twofold symmetric, like a 1D array of cells that is extended perpendicular to the elongation axis. (a2) and (a3) show the alternative elongation axis perpendicular to one of the edges. In these cases there are four long edges competing to absorb membrane proteins. The possible configurations are now fourfold, two of which are polarized perpendicular, and the other two are polarized parallel to the axis of elongation; they are shown in (a1) and (a2), respectively. The latter is destabilized by the nonlocal cytoplasmic interactions. (b) For different values of λ/ℓ0, the magnitude of cooperative self-interactions αs is plotted as a function of L/ℓ0. (c) The angle between the average polarization and the axis of elongation, as a function of the average elongation index E (initial condition and geometry are held fixed). At E≃0.1, the polarization and elongation axis are almost orthogonal; |Φp − Φe| ≃ 87°. (TIFF) [file pcbi.1007454.s007.tiff]

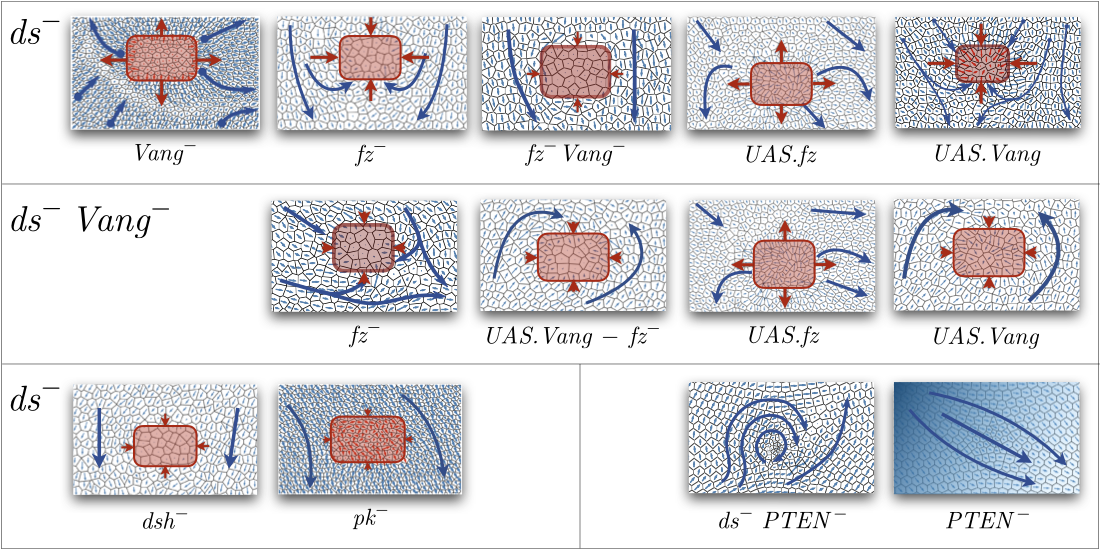

Supplement: S7 Fig — Illustrations of type I, II, and III mutants. The layout of the table is the same as that in the Main Text, and the red arrows show the directions of distortion with respect to the wild-type polarity. This table facilitates a more detailed comparison of the autonomous effects that were absent in Fig (6) of the Main Text. In particular note the differences between the polarities within the putative dsh− and pk− clones, that were induced by small (α, β) and small λ, respectively. (TIFF) [file pcbi.1007454.s008.tiff]
